# *CASCADES*, a novel *SOX2* super‐enhancer‐associated long noncoding RNA, regulates cancer stem cell specification and differentiation in glioblastoma

**DOI:** 10.1002/1878-0261.13735

**Published:** 2024-09-25

**Authors:** Uswa Shahzad, Marina Nikolopoulos, Christopher Li, Michael Johnston, Jenny J. Wang, Nesrin Sabha, Frederick S. Varn, Alexandra Riemenschneider, Stacey Krumholtz, Pranathi Meda Krishnamurthy, Christian A. Smith, Jason Karamchandani, Jonathan K. Watts, Roel G. W. Verhaak, Marco Gallo, James T. Rutka, Sunit Das

**Affiliations:** ^1^ Faculty of Medicine, Institute of Medical Science University of Toronto Canada; ^2^ Arthur and Sonia Labatt Brain Tumor Research Center Hospital for Sick Children Toronto Canada; ^3^ Charbonneau Cancer Institute, Alberta Children's Hospital Research Institute (ACHRI), Department of Biochemistry and Molecular Biology, Cumming School of Medicine University of Calgary Canada; ^4^ Program for Genetics and Genome Biology Hospital for Sick Children Toronto Canada; ^5^ The Jackson Laboratory for Genomic Medicine Farmington CT USA; ^6^ RNA Therapeutics Institute University of Massachusetts Medical School Worcester MA USA; ^7^ Montreal Neurological Institute McGill University Health Center (MUHC) Montreal Canada; ^8^ Division of Neurosurgery, St. Michael's Hospital and Li Ka Shing Knowledge Institute University of Toronto Toronto Canada

**Keywords:** cancer stem cells, cell differentiation, enhancers, long noncoding RNA, *SOX2*, super‐enhancers

## Abstract

Glioblastoma is the most common primary malignant brain tumor in adults, with a median survival of just over 1 year. The failure of available treatments to achieve remission in patients with glioblastoma (GBM) has been attributed to the presence of cancer stem cells (CSCs), which are thought to play a central role in tumor development and progression and serve as a treatment‐resistant cell repository capable of driving tumor recurrence. In fact, the property of “stemness” itself may be responsible for treatment resistance. In this study, we identify a novel long noncoding RNA (lncRNA), cancer stem cell‐associated distal enhancer of *SOX2* (*CASCADES*), that functions as an epigenetic regulator in glioma CSCs (GSCs). *CASCADES* is expressed in isocitrate dehydrogenase (IDH)‐wild‐type GBM and is significantly enriched in GSCs. Knockdown of *CASCADES* in GSCs results in differentiation towards a neuronal lineage in a cell‐ and cancer‐specific manner. Bioinformatics analysis reveals that *CASCADES* functions as a super‐enhancer‐associated lncRNA epigenetic regulator of *SOX2*. Our findings identify *CASCADES* as a critical regulator of stemness in GSCs that represents a novel epigenetic and therapeutic target for disrupting the CSC compartment in glioblastoma.

## Graphical abstract

In glioblastoma, the novel lncRNA CASCADES is enriched in cancer stem cells and regulates stemness by acting as a super‐enhancer‐associated epigenetic regulator of SOX2. Knockdown of CASCADES induces glioma stem cell differentiation towards a neuronal lineage, highlighting its role in treatment resistance and tumor recurrence. CASCADES emerges as a promising therapeutic target for disrupting the cancer stem cell compartment in glioblastoma.

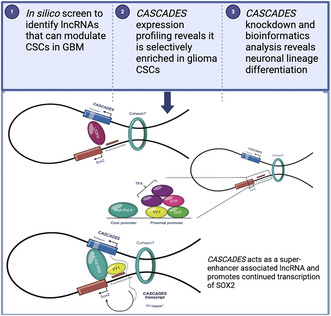

**Abbreviations**
ASOs: antisense oligonucleotides
CASCADES: cancer stem cell‐associated distal enhancer of SOX2
CSCs: cancer stem cells
ESCs: embryonic stem cells
GBM: glioblastoma
GSCs: glioma stem cells
hESCs: human embryonic stem cells
HFNS: human fetal neural stem cells
HUVECs: human umbilical vascular endothelial cells
IDH: isocitrate dehydrogenase
iPSCs: induced pluripotent stem cells
lnc‐eRNAs: enhancer‐associated lncRNAs
lncRNA: long noncoding RNA
RNA Pol II: RNA polymerase II
SE: super‐enhancers
SE‐RNAs: super‐enhancer RNAs
SOX2: sex determining region Y‐box 2
TF: transcription factors

## 1 Introduction

Glioblastoma is the most common primary malignant brain tumor in adults [1]. While standard treatment with aggressive multimodal therapy [2] often results in a brief remission, patients uniformly succumb to disease recurrence and progression, with a two‐year survival rate of < 18% [3].

This pattern of relapse and progression suggests the presence of a subpopulation of cancer cells within the initial tumor that can survive cytotoxic therapies and drive tumor recurrence. Multiple studies support the hypothesis that these cells, defined as cancer stem cells (CSCs), are responsible for glioblastoma recurrence [4, 5, 6, 7]. According to the CSC hypothesis, tumor cells are heterogeneous and hierarchically organized in a manner that recapitulates normal development [7, 8, 9]. Further, the CSC hypothesis posits that tumor growth relies on the proliferation of more differentiated cells arising from the CSC population, in a manner analogous to normal cell systems [9, 10, 11, 12]. In glioblastoma, glioma CSCs (GSCs) have been found to preferentially survive and be enriched by chemoradiation, suggesting that they may serve as a cell repository for tumor recurrence [5, 12, 13, 14].

The regulatory mechanisms that support CSC function are multifaceted and often overlap with mechanisms relevant to normal development. Multiple studies have identified long noncoding ribonucleic acids (lncRNAs) as key drivers in cell identity. LncRNAs are non‐protein coding transcripts longer than 200 nucleotides that have been shown to regulate processes critical to development and cancer progression, acting as both transcriptional activators and repressors [15, 16, 17, 18, 19, 20]. In normal development, lncRNAs play an important role in mediating pluripotency, self‐renewal, and differentiation programs in embryonic stem cells (ESC) [15, 16, 21]. In CSCs, aberrant expression of lncRNAs has been implicated in tumorigenesis, cancer progression, and chemoresistance [22]. In glioblastoma specifically, lncRNAs promote GSC maintenance and confer chemoresistance via several different mechanisms, including the modulation of SOX2, a master regulator of stemness [7, 23, 24, 25, 26].

Among the many mechanisms of action ascribed to lncRNAs, enhancer activity is an important function, primarily because enhancers are capable of driving lineage‐specific gene expression [17, 19, 27, 28]. This function is exemplified by a subset of lncRNAs, known as enhancer‐associated lncRNAs (lnc‐eRNAs), characterized by active enhancer states like H3K4me1 and H3K27Ac [28, 29]. Lnc‐eRNAs can modulate gene expression both in *cis* and *trans* through chromatin looping, and can dictate temporal and spatial gene regulations during development, differentiation, and cell specification events [17, 28].

Super‐enhancers (SEs) are groups of enhancer clusters in close genomic proximity that are enriched in chromatin features characteristic of enhancers, including transcription factors (TFs), chromatin regulators, co‐activators, mediators, RNA polymerase II (RNA Pol II), and enhancer‐associated chromatin marks (especially H3K27ac) [30, 31, 32]. SEs are hierarchically organized, containing both hub and non‐hub enhancers, based on local chromatin landscape [31]. The individual enhancers within SEs can function either independently or synergistically as part of a larger transcription‐regulating complex to promote incremental expression of their associated genes, namely genes crucial for cell fate determination [30, 33]. This feature lends SEs their unique ability to regulate genes with cell‐ and state‐specific roles, making them especially important in maintaining cancer cell identity due to their ability to drive the transcription of genes involved in oncogenic processes [33, 34].

Super‐enhancer RNAs (SE‐RNAs) are a class of noncoding RNAs that are transcribed from super‐enhancer regions [35]. Recent studies have suggested that SE‐RNAs are functionally important in lineage specification and differentiation processes [35, 36]. Notably, SE‐RNAs have been shown to promote glioma progression and to mediate glioma sensitivity to temozolomide [37, 38].

Given their established role in normal stem cell biology and emerging roles in oncogenesis, we hypothesized that lncRNAs could be critical to GSC maintenance in glioblastoma. Here, we report our discovery of *CASCADES*, a novel SE‐associated lncRNA that maintains GSC identity through epigenetic regulation of *SOX2*.

## 2 Materials and methods

All the experiments involving primary human cell lines were generously donated by the laboratory of Peter Dirks in the Arthur and Sonia Labatt Brain Tumor Research Centre and were performed in accordance with the Declaration of Helsinki and well as the research ethics guidelines of the Research Institute at the Hospital for Sick Children, University of Toronto (REB # 0020020238). As described previously [39], all samples were obtained following written informed consent from patients. The cell lines used in this study were received from August to September 2014. All experiments were performed on cells confirmed to be mycoplasma‐free via regular PCR testing every 3 months. GSC lines were confirmed to match their parental primary GBM tumor tissue by microsatellite profiling (The Centre for Applied Genomics, Hospital for Sick Children) or short tandem repeat profiling (Department of Pathology and Laboratory Medicine, University of Calgary) by the laboratory of Peter Dirks within the same timeframe as this paper's experiments. Results from this profiling and cell line characterization have been published [39, 40].

### 2.1 Cell culture

GliNS1 (RRID:CVCL_DG65) and Gli489 (BioSample ID: SAMN06562251) were cultured and expanded on plates coated with 0.01% poly‐L‐ornithine (Sigma, St.Louis, MO, USA, Cat # P4957) followed by 1% laminin (Sigma, Cat # L2020) in phosphate‐buffered saline (PBS) solution. Briefly, the cells were thawed at room temperature and combined with 10 mL of Dulbecco's Modified Eagle Medium with F12 (DMEM/F12) (Wisent, Montreal, Quebec, Canada, Cat #219‐095‐XK) in a 15 mL conical tube. By centrifuging at 800 rpm for 3 min, the cells were pelleted; the supernatant was aspirated, and the cell pellet was resuspended in 10 mL of neural stem cell (NSC) culture media and plated onto laminin‐coated plates. The NSC media was composed of Neurocult NS‐A basal medium human (Stem Cell Technologies, Vancouver, British Columbia, Canada, Cat #05750) along with L‐glutamine (Sigma, Cat #G7513), antibiotic/antimycotic (Sigma, Cat #A5955), bovine serum albumin, epidermal growth factor, basic fibroblast growth factor, B27 supplement, N7 supplement, and heparin. The media was changed every 3–4 days, and the cells were passaged upon reaching ~ 80% confluence.

### 2.2 Astrocyte differentiation

HFNS 6562 (RRID:CVCL_C8ZT), HFNS 7450 (RRID:CVCL_C8ZU), Gli432 (RRID: CVCL_C8ZM), and Gli489 were cultured and expanded on plates coated with 0.01% poly‐L‐ornithine (Sigma, Cat # P4957) followed by 1% laminin (Sigma, Cat # L2020) in phosphate‐buffered saline solution, using normal NSC media. The NSC media was composed of Neurocult NS‐A basal medium human (Stem Cell Technologies, Cat #05750) along with L‐glutamine (Sigma, Cat #G7513), antibiotic/antimycotic (Sigma, Cat #A5955), bovine serum albumin, epidermal growth factor, basic fibroblast growth factor, B27 supplement, N7 supplement, and heparin. The cells were then cultured onto plates coated with Dulbecco's Modified Eagle Medium with F12 (Wisent, Cat #219‐095‐XK) containing gelatin (10 mg·mL^−1^) (Geltrex, Gibco, Fisher Scientific, Waltham, MA, USA, Cat #A14132‐02). Once the cells were 60–70% confluent, they were treated with NSC differentiation media (Neurocult NSA‐A basal medium containing L‐glutamine, antibiotic/antimycotic, and bovine serum albumin along with 1% fetal bovine serum). Four weeks later, the cells were collected for RNA and protein extraction. The controls were cultured on poly‐ornithine and laminin‐coated plates and were treated with normal neural stem cell media.

### 2.3 3′ and 5′ rapid amplification of cDNA ends (RACE)

The 3′RACE (Invitrogen, Waltham, MA, USA, Cat # 18373‐019) and 5′RACE (Invitrogen, Cat #18374‐058) were performed using kits and following manufacturer's instructions. The gene‐specific primers (GSP) were designed separately using oligo primer analysis software (version 7, Molecular Biology Insights, Colorado Springs, CO, USA). For 3′ RACE, first strand cDNA synthesis was performed in glioma stem cells (GliNS1), human fetal neural stem cells (HFNS 5250, RRID:CVCL_C8ZV), and human umbilical vein endothelial cells (HUVEC, RRID:CVCL_B7UI), followed by the amplification of target cDNA using first GSP (GSP1). The 1^st^ amplification product was purified using PureLink PCR Purification Kit (Invitrogen, Cat #K3100), following manufacturer's instructions, and was visualized on a gel. The purified 1^st^ amplification product was then amplified using a nested primer (GSP2), and the 2^nd^ amplification product was purified again, visualized on a DNA gel, and subsequently cloned. On the other hand, for 5′RACE first strand cDNA for 5′RACE was synthesized from GliNS1 using GSP1. The cDNA product was then purified, and a dC‐tail was added to this product. Then, nested amplification was performed using GSP2. An additional nested amplification was performed, if needed, using GSP3. The DNA products were purified after each amplification and were visualized on DNA gels. The final nested amplification product was used for cloning.

### 2.4 Cloning

The 3′ & 5′RACE products were cloned using TOPO TA Cloning Kit (Invitrogen, Cat #K452‐20), following the manufacturer's instructions. Briefly, the products were ligated using the TOPO vector and incubated at room temperature for 15 min. The ligated products were then transformed into *E. coli* through electroporation and plated on kanamycin‐coated agar plates, followed by incubation at 37 °C overnight. Then 3–5 colonies were incubated in lysogeny broth (LB) medium, along with ampicillin, at 37 °C overnight. The plasmids were isolated using Plasmid Mini Prep Kit (Frogga Bio, Toronto, Canada, Cat #PD300), and products were incubated with EcoRI for restriction digest and visualized on a DNA gel for validation.

### 2.5 siRNA‐mediated knockdown of lncRNAs

The cells were cultured in 6‐well plates, using normal NSC culture medium. Twenty‐four hours before siRNA knockdown was performed, the cells were incubated with NSC culture medium without antibiotic/antimycotic. The siRNAs were designed using Custom RNAi Design Tool by Integrated DNA Technologies (IDT) (Coralville, IA, USA) and were resuspended in Tris‐EDTA (TE) buffer to a final concentration of 20 μm. For each well, 2 μL of siRNA was combined with 138 μL of Opti‐Mem Reduced Serum Medium with GlutaMax Supplement (Life Technologies, Carlsbad, CA, USA, Cat #51985‐034) and incubated at room temperature for 5 min. Meanwhile, 16.5 μL of Oligofectamine Transfection Reagent (Life Technologies, Cat #12252‐011) was combined with 43.5 μL of Opti‐Mem. The diluted siRNA + Opti‐Mem was then combined with Oligofectamine + Opti‐Mem, and incubated at room temperature for 15 min to allow complexes to form. The GliNS1 cells were washed once with Opti‐Mem, and 1.3 mL of Opti‐Mem was added to each well, followed by addition of 200 μL of siRNA+Oligofectamine complex. The cells were then incubated at 37 °C for 4 h, and the media was replaced by NSC culture medium without antibiotics/antimycotics. The RNA was extracted 72 h later for analysis by qRT‐PCR.

### 2.6 ASO design and synthesis

ASOs were designed using the LNCASO webserver (https://iomics.ugent.be/lncaso). ASOs were synthesized as fully PS‐modified 5‐10‐5 MOE gapmers (i.e. 5 nucleotides of 2′‐O‐methoxyethyl‐RNA (MOE), 10 DNA nucleotides, 5 MOE nucleotides) using standard phosphoramidite methods on a Dr.Oligo 48 synthesizer (Biolytic, Freemont, CA, USA). Phosphoramidites and standard reagents were purchased from ChemGenes (Wilmington, MA, USA). Coupling time for MOE nucleotides were extended to 2 min. Oligonucleotides were cleaved and deprotected in concentrated aqueous ammonia at 55 °C for 16 h. ASOs were characterized by LC–MS analysis (Table S1) using an Agilent Q‐TOF instrument and were desalted using Amicon ultrafiltration columns (3‐kDa cutoff). The cells were transfected using ASOs following the similar protocol as siRNA‐mediated knockdowns.

### 2.7 Immunocytochemistry

Briefly, the cells were washed with PBS and fixed with 4% paraformaldehyde (PFA) at room temperature for 10 min. They were then permeabilized using 0.3% Triton‐X100 in PBS at room temperature for 5 min and were washed twice by PBS. The fixed cells were then blocked with 5% BSA in PBS for 1 h at room temperature and were then stained using antibodies against Nestin (Millipore, Burlington, MA, USA, Cat# MAB5326) and Sox2 (Abcam, Cambridge, UK, Cat# ab97959) (stem markers), glial fibrillary acidic protein (GFAP) (Santa‐Cruz, Dallas, Texas, USA, Cat# sc‐6171) (astrocyte marker), beta‐tubulin III (Tuj1) (Abcam, Cat# ab78078), and Olig2 (R&D, Minneapolis, MN, USA, Cat# AF2418) (neuron markers). After incubating with primary antibodies at room temperature for 2 h, the cells were then washed twice with high salt PBS, and once again with regular PBS. They were then incubated with appropriate Alexa fluor secondary antibodies, along with phalloidin, at room temperature for 1 h. The cells were then washed as described above, and mounted with DAPI Vectashield media (Vectashield, Newark, CA, USA, Cat # H‐1500). The slides were then visualized using confocal microscopy (*N* = 3 slides/group, and at least 3–6 different fields of view were imaged). Finally, the number of “positive” cells as well as all cells on a field was counted using ImageJ software and plotted as a percentage of positive cells/total cells.

### 2.8 Western blot analysis

Total cell lysates were prepared by harvesting cells in RIPA lysis buffer (Sigma‐Aldrich, St, Louis, MO, USA) with a protease inhibitor cocktail (Roche Diagnostics, Indianapolis, IN, USA). Protein concentration was determined using the Pierce BCA Protein Assay Kit (Thermo Scientific, Rockford, IL, USA). Protein extracts were mixed with 6X SDS sample buffer (Tris pH 6.8, 1.7% SDS, glycerol and β‐mercaptoethanol), and the cell lysates were resolved on 12% SDS‐polyacrylamide gels of 1.5 mm thickness. Proteins were then transferred onto polyvinylidene Fluoride Transfer Membranes (Pall Corporation, Pensacola, FL, USA) and subsequently blocked with 5% skim milk in TBST (20 mm Tris aminomethane, 150 mm NaCl and 0.05% Tween 20; pH 7.4) for 1 h at room temperature. The membranes were incubated overnight at 4 °C with primary antibodies, then at room temperature for 1 h with the secondary antibodies, either horseradish peroxidase‐conjugated goat anti‐rabbit or anti‐mouse immunoglobulin G antibody (1 : 5000; Cell Signaling Technology), and bound primary antibodies were visualized using Western Lightning Plus‐ECL (PerkinElmer Inc., Waltham, MA, USA). The primary antibodies used in this study were as follows: Nestin (Millipore, Cat# MAB5326), Sox2 (Abcam, Cat# ab97959), glial fibrillary acidic protein (GFAP) (Santa‐Cruz, Cat# sc‐6171), beta‐tubulin III (Tuj1) (Abcam, Cat# ab78078), and Olig2 (R&D, Cat# AF2418), phospho H3 (pSer [10]) (Sigma, 9710S).

### 2.9 LIVE/DEAD staining

Cell viability was assessed using LIVE/DEAD Cell Imaging Kit (488/570) (Thermo Fisher Scientific, Waltham, MA, Canada, Cat# R37601). Following manufacturer's protocol, the cells were kept in their original culture vessels. The contents of LIVE green vial were combined with DEAD red vial to create a 2× stock. Then, equal volumes of 2× stock were added to each well and incubated for 15 min at room temperature. The cells were then immediately visualized on fluorescence microscope by a blinded observer. The images obtained were then given to another blinded observer, who provided the final count of LIVE (green) and DEAD (red) cells for each sample.

### 2.10 Cellular subfractionation

The GliNS1 cells were collected and RNA was extracted, and was partitioned into chromosomal, nuclear, and cystoplasmic fractions, as follows. The cells were washed twice with 10 mL PBS^−/−^ and detached using 1 mL of accutase and incubate at 37 °C for 5 min. GliNS1 were then collected with DMEM/F12, and added to 10 mL conical (Falcon, Corning, New York, USA) tube, and pelleted by centrifugation at 14 000 rpm for 4 min. The pellet was then resuspended in cold 1×PBS/1 mm EDTA and transferred to a sterile epitube and centrifuged again to pellet cells at 14 000 rpm for 4 min at 4 °C. The supernatant was discarded, and the wash was repeated once. The pellet was then resuspended in ice‐cold 100 μL lysis buffer (10 mm Tris–HCl, pH 7.5; 150 mm NaCl; 0.15% Nonidet P‐40), and lysed for 5 min on ice. The lysate was layered on top of 2.5 volumes of chilled sucrose cushion (24% sucrose in 10 mm Tris–HCl and 150 mm NaCl), and centrifuged at 14 000 rpm for 10 min at 4 °C. The supernatant, which contained the cytoplasmic fraction, was treated with proteinase K for 1 h at 37 °C, followed by RNA extraction and purification using QIAGEN (Venlo, Netherlands) RNeasy Kit. The pellet was retained on ice until ready for further subfractionation.

The pellet was washed once with ice‐cold 1×PBX/1 mm EDTA, and resuspended in pre‐chilled glycerol buffer 50 μL (20 mm Tris–HCl, pH 7.9, 75 mm NaCl, 0.5 mm EDTA, 0.85 mm DTT, 0.125 mm PMSF, 50% glycerol), by gently flicking tube. Then the equal amount (50 μL) of cold nuclei lysis buffer (10 mm HEPES, pH 7.6, 1 mm DTT, 7.5 mm MgCl_2_, 0.2 mm EDTA, 0.3 m NaCl, 1 m Urea, 1% NP‐40) was added, and vortexed for 2 × 2 s, incubated for 2 min on ice, and then centrifuged for 2 min at 17 530 *g* at 4 °C. The supernatant, which contained soluble nuclear fraction, was treated with proteinase K for 1 h at 37 °C and the RNA was extracted using the QIAGEN RNeasy Kit. The pellet was rinsed with cold 1×PBS/1 mm EDTA, and dissolve in TRIzol (Invitrogen). The RNA was extracted using TRIzol and then purified using the QIAGEN RNeasy Kit. To validate appropriate partitioning of nuclear, chromatin‐bound, and cytoplasmic RNA, the expression of MALAT1 and Xist was evaluated.

### 2.11 Chromatin immunoprecipitation (ChIP) PCR

For chromatin immunoprecipitation, the EZ‐Magna ChIP Kit (Millipore, Cat# 17‐408) was used, following the manufacturer's instructions. Briefly, ~ 20 million cells per ChIP reaction were grown according to the cell culture conditions discussed above. On the day of ChIP, the cells were fixed on culture dish with fresh formaldehyde added directly to the growth media to a final concentration of 1%, and incubated at room temperature for 10 min to allow for crosslinking. The media was removed, and 2 mL of 10× glycine was added to each dish to quench the unreacted formaldehyde and incubated at room temperature for 5 min. The media was aspirated and the cells were washed with cold 1×PBS twice, followed by addition of 2 mL of cold 1×PBS/protease inhibitor (PI). The cells were scraped and centrifuged at 800 ***g*** at 4 °C for 5 min. The supernatant was removed, and the cell pellet was resuspended in 0.5 mL of cell lysis buffer/PI and incubated on ice for 15 min with brief vortexing. The cell suspension was again centrifuged at 800 ***g*** at 4 °C for 5 min to remove the cellular fraction. The nuclei were then lysed with nuclear lysis buffer/PI and sonicated using Diagenode Bioruptor with 30 s on/off cycle for 2 h at 4 °C. The chromatin was sheared to produce fragments of ~ 200 bp in size. The sheared chromatin was then aliquoted, diluted, and 1% volume was collected to serve as an “input” control. To the remaining sheared chromatin, 20 μL of fully suspended protein A magnetic beads, along with 5ug of immunoprecipitating antibody for Rad21 (Abcam, Cat# ab992) or YY1 (Santa‐Cruz, sc‐1703X) was added. For the positive control, an anti‐acetyl histone H3 antibody, and for negative control, the normal rabbit IgG was added and the reactions were incubated for 4 h at 4 °C with rotation. The protein A bead‐antibody/chromatin complex was then washed with the following ice‐cold wash buffers, respectively: low salt immune complex, high salt immune complex, LiCl immune complex, and TE buffer. For each tube, 100 μL of ChIP elution buffer with 1 μL of proteinase K was added, and incubated at 62 °C for 2 h with shaking, followed by incubation at 95 °C for 10 min. The samples were cooled to room temperature, and the DNA was purified using spin columns and was used subsequently for real‐time PCR.

### 2.12 RNA fluorescence *in situ* hybridization

Cells were grown on glass cover slips in 6‐well plates until 70% confluent. Cells were then fixed in 4% cold PFA for 20 min, washed, and hydrated. The cells were hybridized with the human *CASCADES* RNA probe in accordance with the manufacturer's protocols (Advanced Cell Diagnostics, Newark, CA, USA). In brief, cells were pretreated with hydrogen peroxide, permeabilized by incubating with Protease III (1 : 15) for 10 min. After hybridization, a fluorescent kit (version 2) was used to amplify the mRNA signal, and TSA Plus Fluorescein, fluorescent signal was detected using a microscope slide scanner (Olympus, Shinjuku City, Tokyo, Japan) and Confocal microscopy. Representative images were prepared in cell image software (Olympus) and imagej.

### 2.13 Flow cytometry cell cycle analysis

Cells were collected by centrifugation at 400 ***g*** for 5 min at 4 °C. The cells were then spun and resuspended in 50 μL staining medium (HBSS with 2% FBS and azide) and then added to a conical polypropylene tube containing 1 mL of ice‐cold 80% ethanol. The cells were then vortexed and fixed overnight at 4 °C. The fixed cells were then collected by centrifugation at 400 ***g*** for 5 min at 4 °C and washed twice with 1× PBS, then once with staining medium (SM). They were then stained with anti‐Histone H3 [Phospho‐Histone H3 (Ser28) Monoclonal Antibody (HTA28), Alexa Fluor 488, eBioscience] (1 : 500) for 20 min at room temperature, then washed with SM. The pellet was resuspended in 500 μL 2 mg·mL^−1^ RNase A solution and incubated for 5 min at room temperature. Then 500 μL 0.1 mg·mL^−1^ PI solution was added and vortexed to mix. The pellet was incubated for 30 min at room temperature, protected from light, and filtered through Nitex into FACS tubes. Finally, the cells were run through a BD analyzer, and the results were analyzed using FlowJo software.

### 2.14 Bioinformatics analysis

LncRNA expression data was obtained from the Wellcome Trust Ensembl Genome Browser [41]. *CASCADES* (LINC01995) tissue expression data was collected from EMBL‐EBI Expression Atlas (https://www.ebi.ac.uk/gxa/home). The splice variants were identified using NCBI (https://www.ncbi.nlm.nih.gov/gene/?term=LINC01995) and UCSC Genome Browser, whereas the gene conservation analysis was performed using UCSC Genome Browser (https://genome.ucsc.edu/). For enhancer analysis, the WashU Epigenome Browser (https://epigenomegateway.wustl.edu/browser/) [42] was used, with tracks for RAMPAGE‐Seq data on NSC and neural cells.

### 2.15 IDH mutation analysis

Raw RNAseq fastq files from initial and recurrent glioma pairs were downloaded from the European Genome‐Phenome Archive under accession numbers EGAS00001001033 and EGAS00001001880, as well as the Genomic Data Commons Legacy Archive GBM and LGG datasets (https://portal.gdc.cancer.gov/legacy‐archive) [43, 44, 45]. Together, the samples used for these analyses comprise the publicly available expression data for the glioma pairs that are part of the Glioma Longitudinal Analysis (GLASS) Consortium (https://www.synapse.org/#!Synapse:syn17038081/). Each fastq file was preprocessed using fastp v0.20.0 and then input into kallisto v0.46.0 using Ensembl v75 noncoding RNA as the reference index. Transcript per million (TPM) values output by kallisto was used for downstream analyses. The sample sizes for each group are as follows: initial IDH mut (*n* = 10), initial IDH wt (*n* = 31), recurrent IDH mut (*n* = 10), and recurrent IDH wt (*n* = 31). Both the initial and recurrent tumors come from the same patients, so the numbers are the same for each IDHmut/IDHwt category.

### 2.16 Hi‐C

Hi‐C contact matrices, loop calls, H3K27ac ChIP peaks, and CTCF ChIP peaks from primary glioblastoma cultures were accessed from GEO accession GSE121601 and https://wangftp.wustl.edu/hubs/johnston_gallo/ [46]. Two‐dimensional DNA contact matrices were displayed using Juicebox 1.11.08 [47] at 5‐kb resolution with balanced normalization and with identical color range applied to all panels. One‐dimensional tracks were displayed using IGV [48].

### 2.17 RNA‐Seq

RNA was extracted using RNeasy kit (Qiagen). For ribosomal depletion, 1–2 mg of total RNA was used for Ribo‐Zero rRNA Removal kit (Illumina, San Diego, CA, USA) according to the manufacturer's protocol. Ribosomal‐depleted library was processed for TruSeq Stranded Total RNA sequencing according to the manufacturer's protocol (Illumina). The quality and quantity of total RNA and final libraries were assessed using Agilent Technologies 2100 Bioanalyzer. RIN (RNA Integrity Number) of > 9 was used for the sequencing experiments.

### 2.18 Data analysis

For RNA‐Sequencing data, reads were assessed for sequencing quality using fastqc (v.0.11.7, Babraham Institute, Cambridge, UK). Paired‐end reads were aligned and counted using star (v.2.6.0c, Cold Spring Harbor Laboratory, Cold Spring Harbor, New York, USA). STAR genome index was generated using GRCh38.p13 assembly and GRCh38.101 GTF from Ensembl. Raw counts were pre‐filtered on the basis on row‐wise sums across all samples with a cutoff of 5. Differential expression analysis was computed using deseq2 (v1.28.1, Dana Farber Cancer Institute, Boston, MA, USA). The network map was generated using the BioBox Platform (https://biobox.io), a cloud genomic data analytics platform.

## 3 Results

**Fig. 1:**
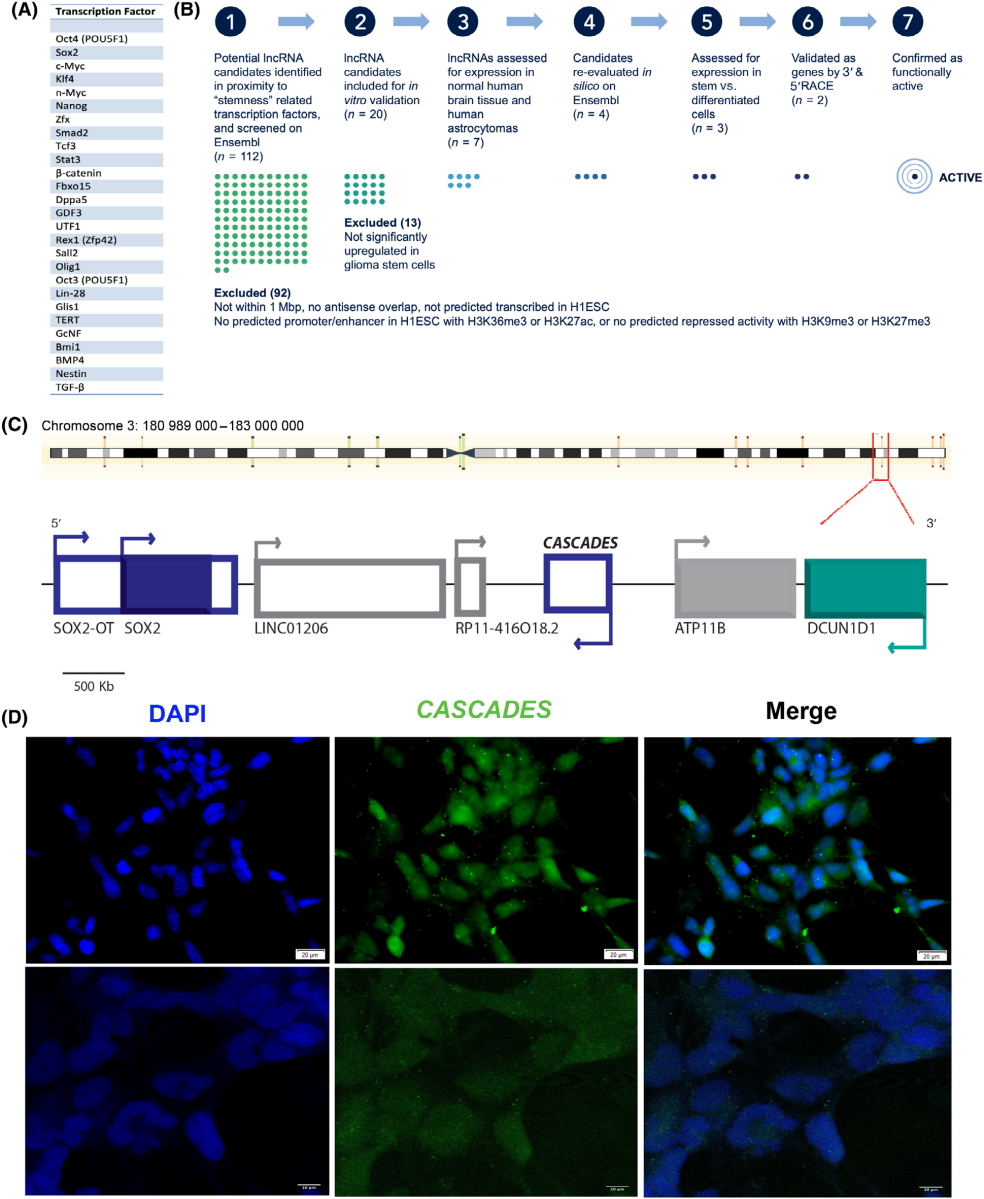
Long noncoding RNA screen to identify novel lncRNA targets relevant to glioma biology. (A) List of transcription factors used in the *in silico* long noncoding (lncRNA) screen. (B) Pipeline for *in silico* screen to identify novel lncRNAs. (C) Genomic map depicting the location of *CASCADES* lncRNA in relation to *SOX2*. (D) RNA *in situ* hybridization of *CASCADES* in glioma stem cell (GliNS1) cell line, with results replicated 3 times per group. Scale bars 20 μm (top) and 10 μm (bottom).

To identify lncRNAs that could modulate GSC identity, we employed an *in silico* “nearest‐neighbor” approach [18, 49] for lncRNA candidates in proximity to transcription factors that have been implicated in regulating self‐renewal or pluripotency of ESCs, that have been used to reprogram somatic cells into induced pluripotent stem cells (iPSCs) or to reprogram GSCs, including: POU5F1 (Oct4), Sox2, β‐catenin (CTNNB1), Myc, Nanog, Klf4, Zfx, Smad2, Tcf3, Stat3, Fbxo15, GDF3, UTF1, Rex1 (Zfp42), Sall2, and Olig1 [15, 50, 51] (Fig. 1A). Using the Ensembl Genome Browser from the European Bioinformatics Institute and the Wellcome Trust Sanger Institute [41], we then evaluated the genomic loci of these transcription factors. We found 112 putative lncRNAs in proximity to these genes of interest (GOI). These lncRNAs were interrogated further using data cataloged in Ensembl, including predicted expression in different cell lines and differential chromatin marks within the genomic loci.

Following this initial screen, we interrogated lncRNA candidates using the following criteria: (a) the lncRNA must be within 1 Mbp either upstream or downstream from a GOI; (b) the lncRNA must not have any antisense overlap with the GOI; (c) the lncRNA must be predicted to be transcribed in at least one human embryonic stem cell line (H1ESC); (d) the gene locus containing the lncRNA must have a predicted promoter or enhancer in H1ESC with either H3K36me3 or H3K27ac histone features, or the gene locus must show repressed activity with H3K9me3 or H3K27me3 histone marks. Using this approach, we identified a list of 20 lncRNAs for further study (Table S2). We then validated the expression of the 20 selected lncRNAs in human embryonic stem cells (hESCs), induced pluripotent stem cells (iPSCs), human umbilical vascular endothelial cells (HUVECs), human fetal neural stem cells (HFNS), and GSCs. Based on these findings, we chose to study an intergenic lncRNA found on chromosome 3 at 3q26.33 (gene ID: LINC01995), downstream from *SOX2* and on the opposite strand, which we named Cancer Stem Cell‐Associated Distal Enhancer of *SOX2* (*CASCADES*; Fig. 1B,C).

**Fig. 2:**
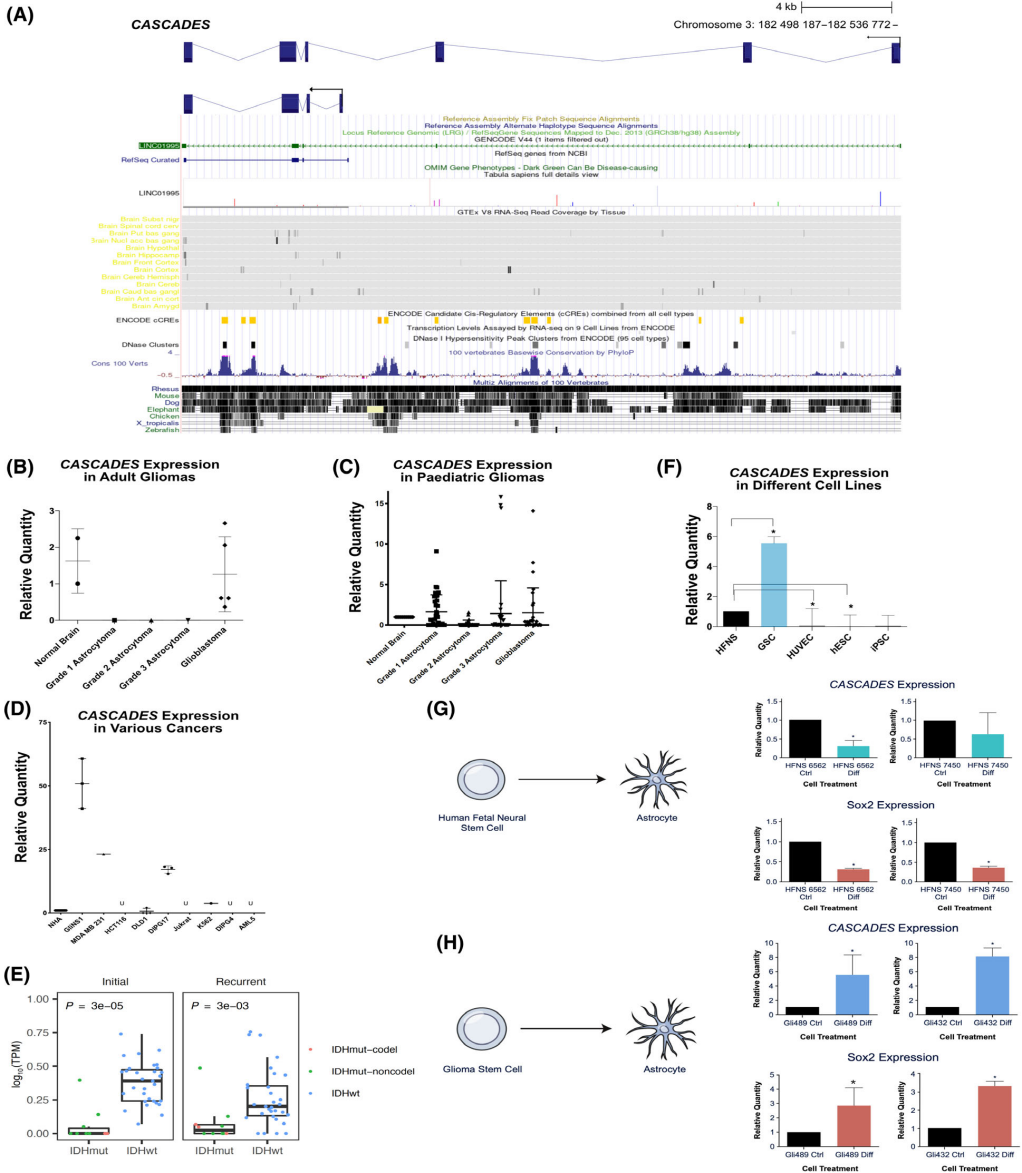
Expression profile of Cancer Stem Cell‐Associated Distal Enhancer of SOX2 (*CASCADES*). (A) University of California Santa Cruz (UCSC) Genome Browser tracks show that both of the *CASCADES* transcripts have peaks conserved across many different species. (B) In adults, *CASCADES* is expressed in the normal brain and grave IV gliomas (glioblastoma) only. Error bars represent standard deviation (SD). (C) In pediatric population, *CASCADES* is expressed in all grades of gliomas. Error bars indicate SD. (D) *CASCADES* is expressed in gliomas, breast cancer (cell line MDA MB 231), colorectal cancer (cell line DLD1), diffuse intrinsic pontine glioma (cell line DIPG17), and chronic myelogenous leukemia (cell line K592). Error bars indicate SD. (E) RNA‐seq of both primary and recurrent gliomas from the Glioma Longitudinal Analysis (GLASS) shows that *CASCADES* is significantly enriched in IDH‐wildtype compared to IDH‐mutant tumors. Differences were assessed using ANOVA; error bars indicate SD. (F) *CASCADES* is enriched in glioma stem cells (GSCs) vs. normal fetal neural stem cells (HFNS), human umbilical vein endothelial cells (HUVECs), human embryonic stem cells (hESCs), and induced pluripotent stem cells (iPSCs) (*P < 0.05, evaluated using ANOVA). Error bars indicate SD. (G) Upon differentiation of human fetal neural stem cells into astrocytes, *CASCADES* decreased in expression (*P < 0.05, evaluated using ANOVA). Error bars indicate SD. (H) When glioma stem cells are forced to differentiate into astrocyte‐like cells, *CASCADES* expression is enriched (*P < 0.05, evaluated using ANOVA). Error bars indicate SD.

Results of RNA *in situ* hybridization and 3′ and 5′ RACE validated *CASCADES* as a transcribed gene (Fig. 1D; Fig. S1). Study of *CASCADES* using the UCSC Genome Browser revealed a gene with two common splice variants that was highly conserved across different species (Fig. 2A). Analysis using the EMBL‐EBI Expression Atlas suggested that *CASCADES* is expressed mostly in the testes and the developing brain (Fig. S2A,B). In adults, *CASCADES* is expressed in normal brain and GBM, with no detectable expression (by qPCR) in adult isocitrate dehydrogenase (IDH)‐mutant low‐grade astrocytomas (Fig. 2B). In contrast, we found evidence of *CASCADES* expression in all IDH‐wild‐type pediatric‐type gliomas, regardless of grade (Fig. 2C). In addition, we found *CASCADES* expression in breast cancer (MDA MB 231), colorectal cancer (DLD1), diffuse intrinsic pontine glioma (DIPG17), and chronic myelogenous leukemia (K592) (Fig. 2D).

To clarify the nature of its differential expression in glioma, we interrogated *CASCADES* expression using RNA‐sequencing data of both primary and recurrent gliomas from the Glioma Longitudinal Analysis (GLASS) consortium database. These studies showed that in glioma, *CASCADES* is highly expressed in both primary and recurrent gliomas with wild‐type isocitrate dehydrogenase (IDH); conversely, we found no significant evidence of expression of *CASCADES* in IDH‐mutant gliomas, regardless of 1p and 19q deletion status (Fig. 2E). Indeed, the expression of *CASCADES* in pediatric but not adult low‐grade gliomas speaks to the frequency of IDH mutation in the latter, and near absence in the former. These data suggest that *CASCADES* expression occurs in a mutually exclusive fashion with IDH mutation in human glioma. Moreover, bioinformatic analysis of the data from the Cancer Genome.

Atlas revealed that *CASCADES* expression is associated with shorter survival in glioma patients in a dose‐dependent manner (Fig. S2C).

As lncRNAs have been shown to demonstrate both tissue‐ and cell‐specific patterns of compartmentalization, we then turned to studies to determine if *CASCADES* expression showed cell specificity. We found *CASCADES* to be enriched in GSCs versus normal human fetal neural stem cells (hfNSC) (Fig. 2F). Interestingly, forced differentiation of hfNSCs to astrocytes (Fig. S3A,B) resulted in a significant decrease in *CASCADES* expression (Fig. 2G), while forced differentiation of GSCs to astrocyte‐like cells (Fig. S3C,D) resulted in an unexpected increase in *CASCADES* expression (Fig. 2H).

**Fig. 3:**
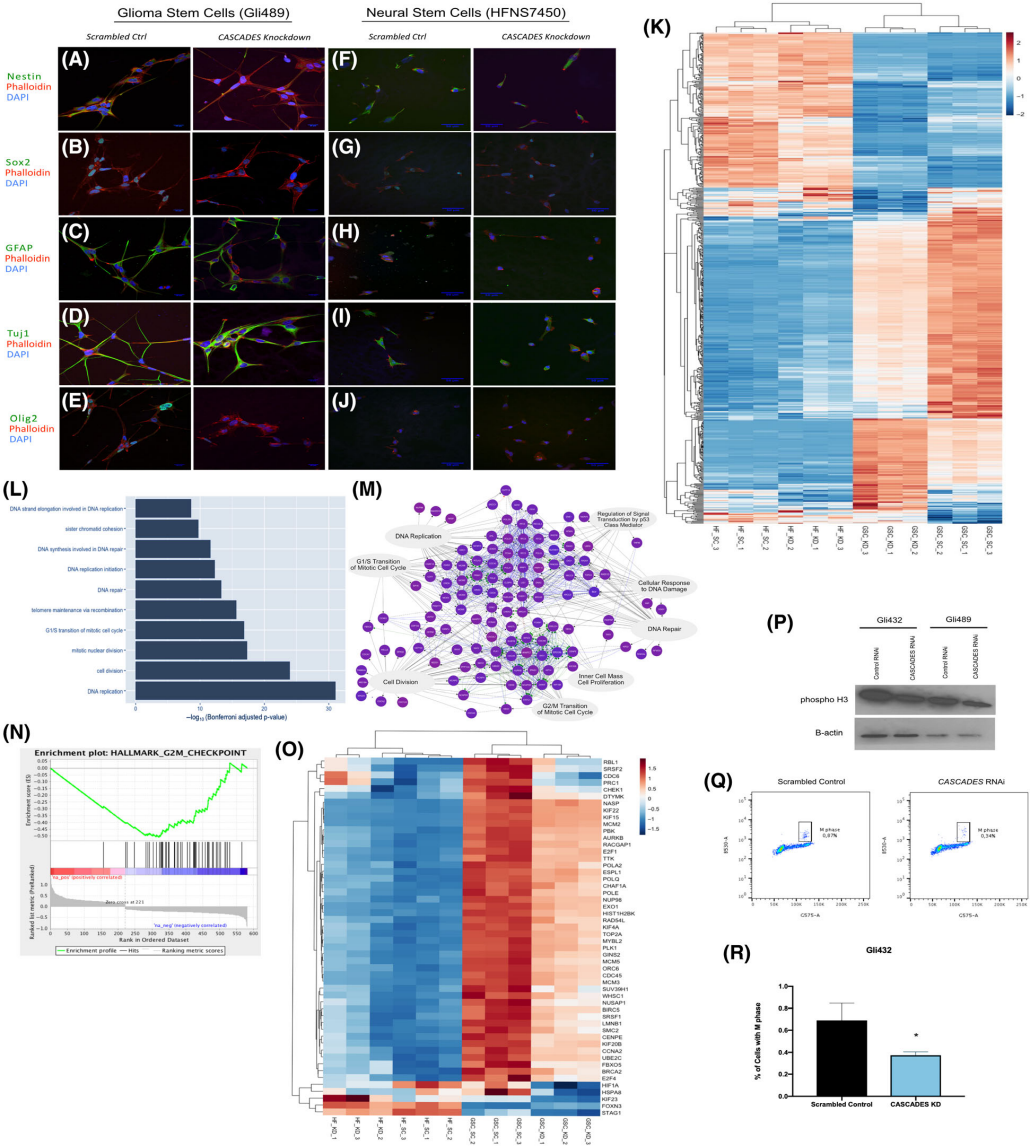
The knockdown of *CASCADES* was performed in glioma stem cells and human fetal neural stem cells (*N* = 3/group) using small interfering RNA (siRNA). (A–C) The stem markers, Nestin and Sox2, as well as glial marker GFAP decreased in expression upon the knockdown of CASCADES in glioma stem cells (GSCs), *n* = 3/group. Scale bar is 25 μm. (D, E) The expression of neural marker, Tuj1, increased upon the knockdown of CASCADES, whereas Olig2 decreased. Scale bar is 25 μm. (F, G) The knockdown of CASCADES in human fetal neural stem cells (HFNS) decreased the stem markers, Nestin and Sox2. Scale bar is 25 μm. (H–J) The expression of glial and neural markers, GFAP, Tuj1, and Olig2, respectively, was unchanged upon knockdown of CASCADES. Scale bar is 25 μm. (K) RNA‐Sequencing analysis showed differential gene expression upon knockdown of CASCADES only in GSCs, whereas this effect was not as pronounced in HFNS (*n* = 3/group). (L) Gene Ontology of the top 10 pathways perturbed upon the knockdown of CASCADES. (M) Gene Set Enrichment Analysis of the differentially expressed genes upon the knockdown of CASCADES in GSCs revealed pathways involved in regulation of cell cycle, mitosis, as well as DNA repair pathways. (N) Gene Set Enrichment Analysis of the differentially expressed genes upon the knockdown of CASCADES in GSCs revealed genes involved in the G2/M check point of the cell cycle. (O) The knockdown of CASCADES results in downregulation of genes involved in the G2/M checkpoint only in GSCs, whereas this effect is unobserved in HFNS (*n* = 3/group). (P) The expression of cell mitosis marker, phospho H3, decreases upon the knockdown of CASCADES in GSCs. (Q, R) Cell cycle analysis using histone H3 as a marker showed a significant reduction of the cells in M phase of the cell cycle upon knockdown of CASCADES in GSCs, compared to scrambled control (*P < 0.05, evaluated using ANOVA). Data shown in R represent *n* = 3/group with error bars indicating standard deviation.

In order to understand the functional role of *CASCADES* in GSCs, we performed siRNA‐mediated knockdown *in vitro* using two previously validated primary human GSC lines, GliNS1 and Gli489 (*N* = 3/group; Fig. S3E,F). *CASCADES* knockdown in GSCs resulted in a significant decrease in *CASCADES* expression, compared to universal (scrambled) control. *CASCADES* knockdown had no adverse effects on cell viability, as assessed using LIVE/DEAD staining and the trypan blue exclusion assay (Fig. S3G–J), but did result in a significant decrease in the stemness markers, Nestin (Fig. 3A; Figs S4 and S5) and Sox2 (Fig. 3B; Figs S4 and S5), in comparison to their respective scrambled controls. Interestingly, the expression of the astrocyte marker, GFAP, also significantly decreased after knockdown of *CASCADES* (Fig. 3C; Figs S4 and S5). Conversely, CASCADES knockdown resulted in a significant increase in the expression of the neuronal marker, Tuj1, and decrease in the anti‐neurogenic transcription factor, Olig2, compared to scrambled control (Fig. 3D,E; Figs S4 and S5). Similarly, *CASCADES* knockdown in the hfNSC line, HFNS 7450 (*N* = 3/group), resulted in a decrease in Nestin (Fig. 3F; Figs S6 and S7) and Sox2 (Fig. 3G; Figs S6 and S7), compared to scrambled control. However, unlike in GSCs, the expression of GFAP (Fig. 3H; Figs S6 and S7), Tuj1 (Fig. 3I; Figs S6 and S7), and Olig2 (Fig. 3J; Fig. S6) in hfNFCs was unchanged after *CASCADES* knockdown, compared to scrambled controls.

To determine if *CASCADES* knockdown has a broader effect on gene expression, we then performed RNA‐sequencing in GSCs and NSCs before and following siRNA treatment. RNA‐seq analysis performed after *CASCADES* knockdown showed differential cellular gene expression in GSCs only, whereas no such effect was observed upon knockdown of *CASCADES* in NSCs (Fig. 3K). Gene Ontology (GO) analysis and Gene Set Enrichment Analysis (GSEA) suggested that the effect of CASCADES knockdown in GSCs was predominantly on transcriptional programs involved in DNA replication and control of the cell cycle (Fig. 3L,M).

GSEA of differentially expressed genes in GSCs and NSCs following *CASCADES* knockdown also showed GSC‐specific downregulation of genes involved in the G2/M checkpoint (Fig. 3N,O). Indeed, western blot analysis showed a decrease in phosphorylated H3 expression upon knockdown of *CASCADES* in GSCs (Fig. 3P), consistent with decreased mitosis. Similarly, cell cycle analysis in GSCs using histone H3 as a marker for mitosis showed a significant reduction in the proportion of cells in the M phase with *CASCADES* knockdown, compared to scrambled control (Fig. 3Q,R).

**Fig. 4:**
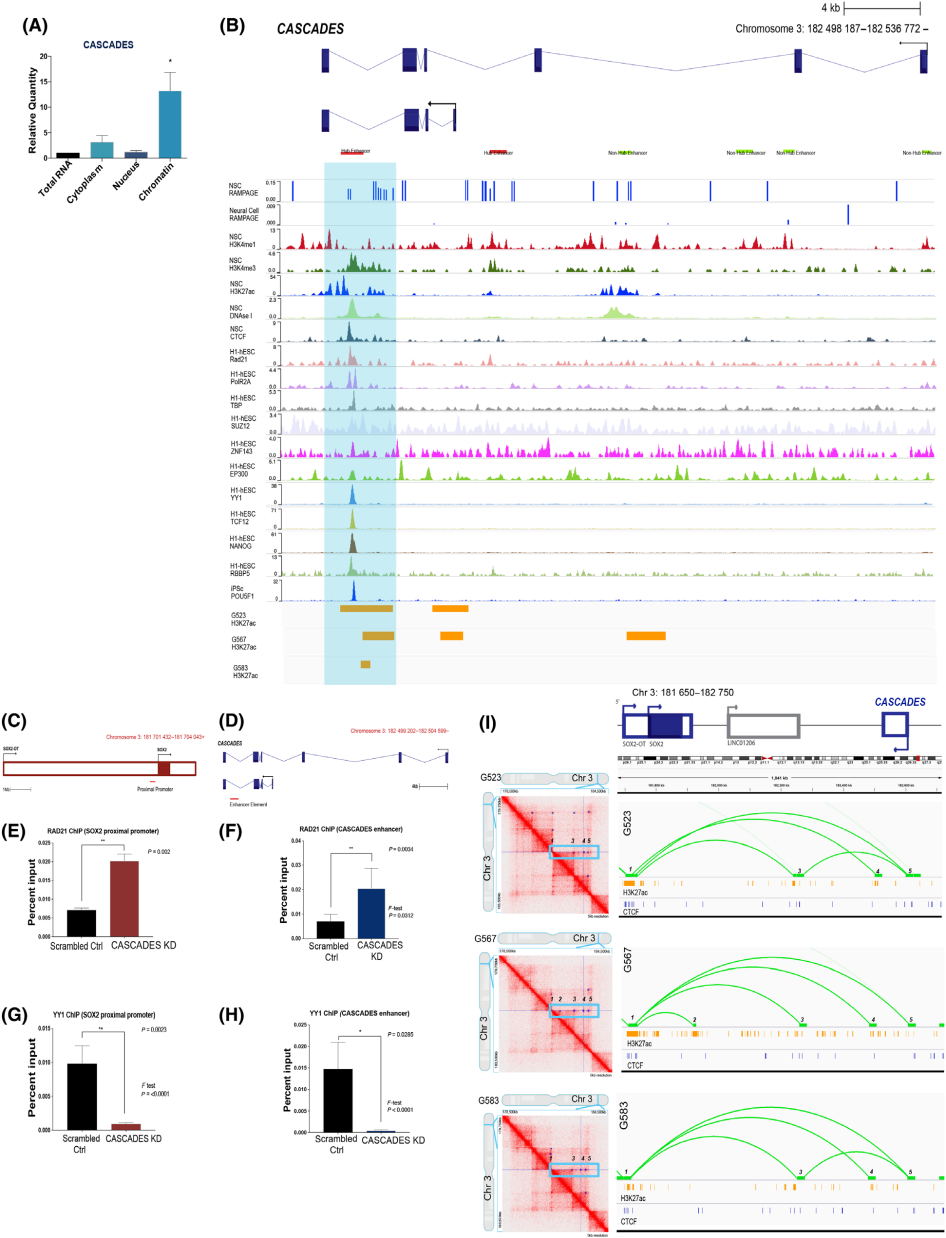
*CASCADES* is a super‐enhancer‐associated lncRNA. (A) Subcellular localization of *CASCADES* in GSCs (*n* = 3) showed that it is mostly chromatin‐bound compared to total RNA (*P < 0.05, differences evaluated using ANOVA). Error bars represent standard deviation (SD). (B) WashingtonU Epigenome Browser tracks in conjunction with RAMPAGE‐Seq data revealed a hub enhancer within the last intron of *CASCADES*, indicated by DNAse I hypersensitive sites, along with open chromatin marks H3K4Me1, H3K4Me3, and H3K27ac. This hub enhancer is conserved across both transcripts of *CASCADES*. The second, longer transcript of *CASCADES* also contains one other open chromatin site and 4 non‐hub enhancers. ChIP‐Seq of H3K27ac in glioma stem cell (GSC) lines G523, G567, and G583 (*n* = 3/group) showed H3K27ac enrichment at the hub enhancer. Red bars indicate open chromatin, whereas green bars indicate non‐hub enhancers. Hub enhancer is highlighted by blue panel. Error bars indicate SD. (C) Location of the Sox2 proximal promoter used for ChIP. (D) Location of the Enhancer Element within CASCADES used for ChIP. (E, F) ChIP‐PCR of Rad21 showed an increased binding of Rad21 at both *SOX2* proximal promoter (**P < 0.01) and the enhancer element found within *CASCADES* (**P < 0.01) upon the knockdown of *CASCADES*. Differences between groups were evaluated using ANOVA. Error bars indicate SD, with *n* = 3/group. (G, H) The knockdown of *CASCADES* results in decrease binding of YY1 at the proximal promoter of *SOX2* (**P < 0.01) and the *CASCADES* enhancer element (*P < 0.05). Differences between groups were evaluated using ANOVA. Error bars indicate SD, with *n* = 3/group. (I) Hi‐C performed in glioma stem cells (*n* = 3) revealed chromatin loops that occur between the SOX2 proximal promoter and the hub enhancer element found within *CASCADES*. ChIP‐Seq of CTCF and H3K27ac in GSC lines also showed enrichment at the contact loop points.

We then sought to determine the mechanism of action of *CASCADES*. First, we performed cellular subfractionation followed by qRT‐PCR to identify the subcellular location of the *CASCADES* transcript. While we found evidence of *CASCADES* transcript in the cytoplasm and nucleus, it was found to be mostly chromatin‐bound (Fig. 4A; Fig. S8A,B). Further analysis of *CASCADES* on the Washington University Epigenome Browser showed DNase I hypersensitive sites within the gene body (Fig. 4B). Bioinformatic analysis of *CASCADES* revealed a stem cell‐specific distal enhancer element for *SOX2* in GSCs. This enhancer element was found within the last intron of *CASCADES* and was conserved across both splice variants. The presence of H3K4me3 and H3K27ac peaks, flanked by H3K4me1, suggested open chromatin and an active hub enhancer (Ensembl ID: ENSR00001076566). In addition, the second, longer transcript of *CASCADES* contained one other active chromatin site (Ensembl ID: ENSR00000710691) and four other non‐hub enhancers (Ensembl IDs: ENSR00000710692, ENSR00000710695, ENSR00000710696, and ENSR00000710698). The neighboring genomic region also contained an enhancer (Ensembl ID: ENSR00000710689), revealing the presence of a super‐enhancer within this region. Taken together, this suggests that *CASCADES* is a super‐enhancer‐associated lncRNA. RAMPAGE‐Seq data showed the presence of transcriptional start site peaks for *CASCADES* in NSCs but not differentiated neurons (Fig. 4B), suggesting a mechanism for its stem cell‐specific function.

The hub enhancer element also showed binding of transcription factors and proteins that are critical to gene transcription, such as Rad21, YY1, TCF12, as well as core transcription factors like NANOG and POU5F1 (Oct4). Furthermore, ChIP‐Seq of H3K27ac in GSC lines (G523, G567, and G583) (Johnston et al. 2019) showed enrichment of H3K27ac at the hub enhancer (Fig. 4B). We then sought to test the hypothesis that *CASCADES* could be exerting its effect via chromatin looping events. We first performed chromatin immunoprecipitation (ChIP) of Rad21, a protein mediating promoter‐enhancer looping. Rad21‐ChIP showed high co‐localization of Rad21 at the *CASCADES* enhancer element and *SOX2* proximal promoter. Furthermore, RNAi of *CASCADES* resulted in decreased binding of YY1 and RNA Pol II at the *SOX2* proximal promoter (Fig. 4C,G; Fig. S9) and *CASCADES* enhancer element (Fig. 4D,H; Fig. S9), and a corresponding increase in Rad21 binding (Fig. 4C–F; Fig. S9). In addition, Chromatin Conformation Capture (Hi‐C) of three GSC lines (G523, G567, and G583) [46] confirmed the presence of chromatin loops between the *CASCADES* enhancer element and *SOX2* proximal promoter (Fig. 4I), further supporting that chromatin looping is important to its mechanism of action. ChIP‐Seq of H3K27ac and CTCF also showed their enrichment at the loop contact points in GSCs.

To determine functional differences between the *CASCADES* transcript and the enhancer elements found within *CASCADES*, we designed antisense oligonucleotides (ASOs) targeting various sites across the *CASCADES* gene. We utilized ASOs to perform knockdown studies, as ASOs are optimal for silencing nuclear, chromatin‐associated targets, such as *CASCADES* [52]. Furthermore, ASOs have been used clinically as central nervous system (CNS) therapeutics [53], making them an attractive option to target a GSC‐specific gene like *CASCADES*.

**Fig. 5:**
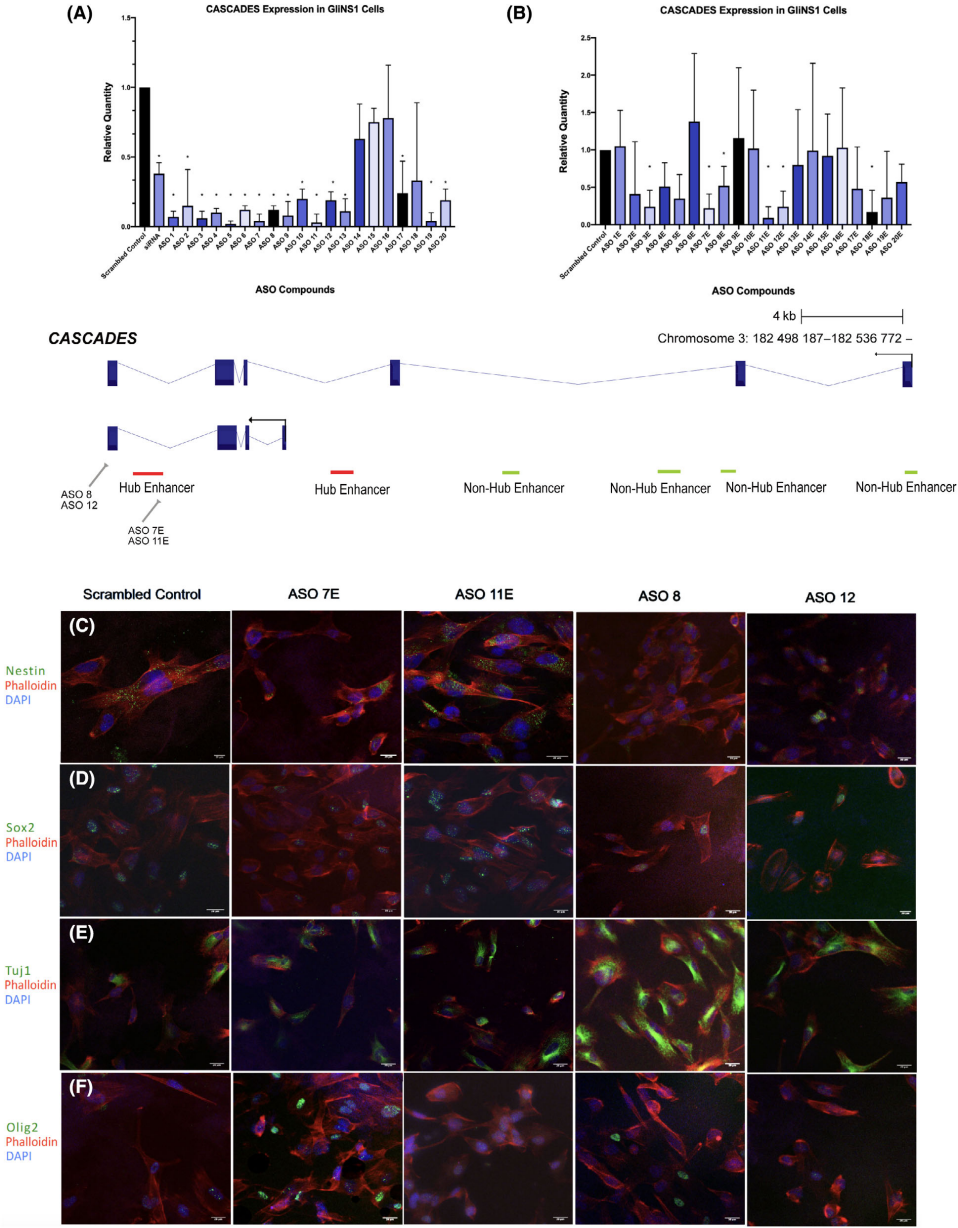
The knockdown of *CASCADES* was performed in glioma stem cells (*N* = 3/group) using ASOs. (A) Antisense oligonucleotides (ASO) were designed to target various sites across the *CASCADES* transcript (bottom panel). The expression of *CASCADES* was determined using qPCR, and ASO compounds 8 and 12 were selected based upon the extent of knockdown, and the proximity to the poly‐A tail. Differences in the relative quantity of each ASO compound compared to scrambled control were evaluated using ANOVA, with significance defined as *P* < 0.05 and denoted by an asterisk, Error bars indicate standard deviation (SD) in the mean of *n* = 3/group. (B) ASO compounds designed to target the hub enhancer element. Compounds 7E and 11E were selected based on the extent of knockdown. Differences in the relative quantity of each ASO compound compared to scrambled control were evaluated using ANOVA, with significance defined as *P* < 0.05 and denoted by an asterisk, Error bars indicate standard deviation (SD) in the mean of *n* = 3/group. (C, D) The stem markers, Nestin and Sox2 decreased in expression upon the knockdown of *CASCADES* in *n* = 3 glioma stem cells (GSCs) using ASO 7E, ASO 8, and ASO 12. Scale bar is 20 μm. (E) The expression of neural marker, Tuj1, increased upon the knockdown of *CASCADES* in ASO 11E, ASO 8, and ASO 12. Scale bar is 20 μm. (F) The expression of antineurigenic marker Olig2 decreased across all ASO compounds used. Scale bar is 20 μm.

We screened 21 ASOs and identified multiple hits with robust knockdown (at least 70% reduction in mRNA expression) of *CASCADES*. ASOs 8 and 12 were designed proximal to the polyadenylation signal to prevent premature transcriptional termination (Fig. 5A) [54, 55]. ASOs 7E and 11E were designed to target the hub enhancer element (Fig. 5B). We treated GSCs (Gli432 and Gli489) with each of the four ASOs to silence the expression of *CASCADES* (*N* = 3/group). Consistent with the siRNA data, knockdown of *CASCADES* using ASOs 7E, 8, and 12 resulted in decreased expression of stem cell markers, Nestin and Sox2 (Fig. 5C,D; Fig. S10A,B). The expression of the neuronal marker, Tuj1, increased upon knockdown using ASOs 11E, 8, and 12 (Fig. 5E; Fig. S10C), whereas Olig2 decreased in expression following treatment with all four ASO compounds (Fig. 5F; Fig. S10D). While the expression of Sox2 reduced upon the knockdown of *CASCADES* using different ASOs, we observed more robust neuronal differentiation using ASOs targeting the 3′ end of the *CASCADES* transcript. These findings show that the *CASCADES* transcript is a crucial component in the mechanism of action for the *SOX2* distal super‐enhancer embedded within this genomic region.

## 4 Discussion

LncRNAs are genetic regulatory elements that, through their role as transcriptional modifiers, can influence global patterns of gene expression in the cells in which they are expressed. As such, lncRNAs have been shown to regulate pluripotency, self‐renewal, and differentiation in embryonic stem cells, and have been shown to be critical as mediators of disease progression and metastasis in multiple cancers. Through a combination of *in silico* and *in vitro* studies, we identified *CASCADES*, a novel lncRNA that regulates stem cell identity in glioblastoma by acting as a *SOX2* super‐enhancer associated lncRNA.

While *CASCADES* is enriched in both NSCs and GSCs, our data shows that the effect of *CASCADES* knockdown on cell cycle pathways is GSC‐specific and promotes GSC differentiation towards a neuronal phenotype. In contrast, no such phenotype is observed upon knockdown of *CASCADES* in NSCs. Notably, *CASCADES* expression is limited to IDH‐wild‐type gliomas. Interestingly, GSC differentiation in IDH‐mutant tumors has been shown to occur towards an astrocytic (AC‐like) or oligogodendrocytic (OPC‐like) cellular state, but not towards a neuronal (NPC‐like) one [6, 56, 57, 58]. Conversely, glioma cells that differentiate towards an NPC‐like cellular state are found in IDH‐wild‐type glioblastomas. It is intriguing to speculate that *CASCADES*, as a lncRNA specific to IDH‐wild‐type tumors, could account for the specificity of the NPC‐like state to IDH‐wild‐type gliomas and acts as an epigenetic regulator specific to the IDH‐wild‐type context.

Bioinformatics analysis of *CASCADES* revealed a cell type‐specific distal enhancer element for *SOX2* in GSCs, which we determined to be a hub enhancer and part of a distal super‐enhancer of *SOX2* from which *CASCADES* is transcribed. Super‐enhancers are important regulatory elements that generate cell type‐specific transcriptional responses. Unsurprisingly, they are central to the maintenance of cancer cell identity because of their role in modulating expression of genes involved in cell growth and lineage specificity [34, 35]. In addition, SEs can promote oncogenic transcription of several growth‐related genes and can be acquired *de novo* during cellular transformation [33, 34]. Moreover, SEs show remarkable tumor type specificity in many different types of cancers, including medulloblastoma [59], neuroblastoma [60], small‐cell lung cancer [61], breast cancer [62], esophageal [63] and gastric cancers [64], and melanomas [34, 65] thus, making them attractive therapeutic targets in cancer [33].

**Fig. 6:**
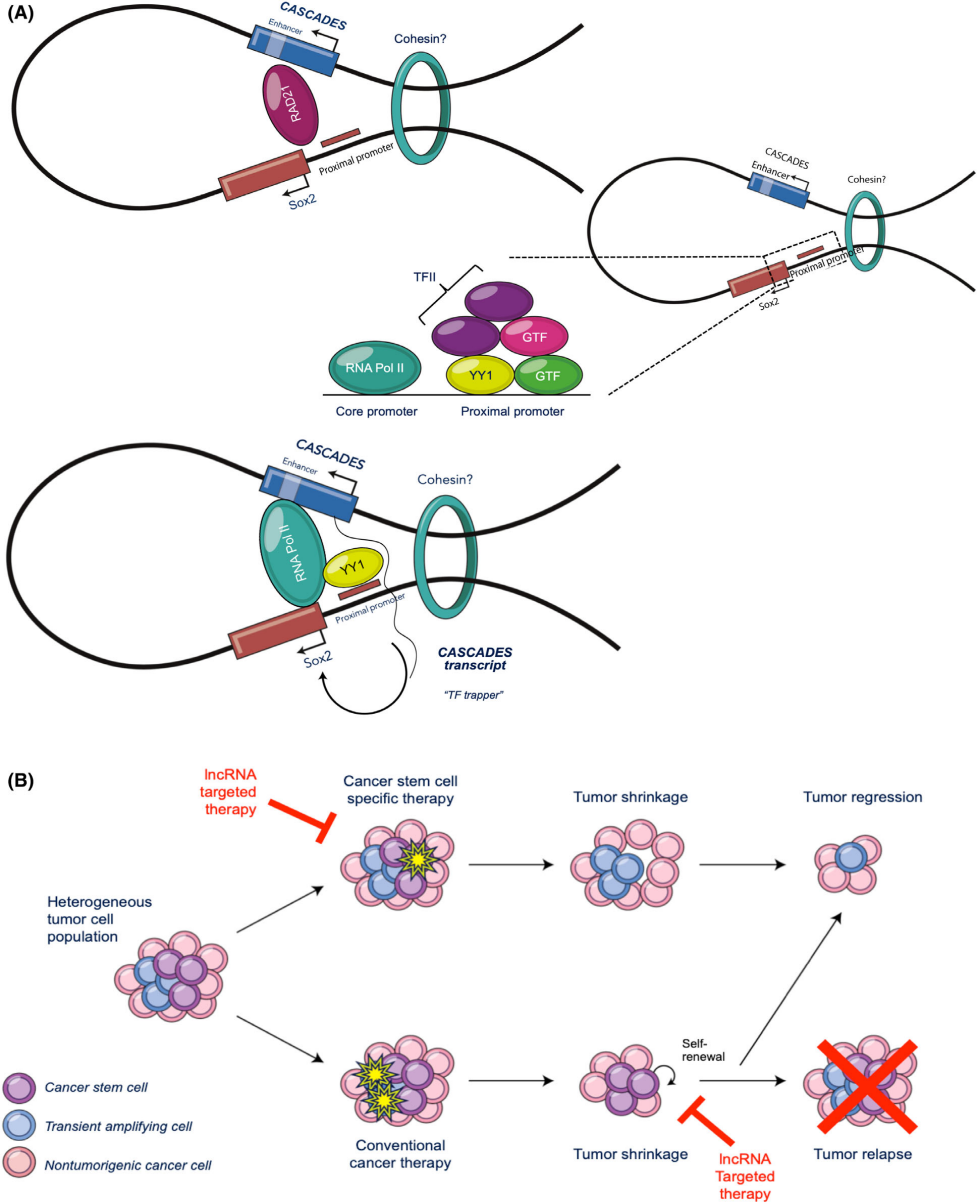
*CASCADES* is a potential epigenetic target for disrupting the CSC niche in GBM. (A) The binding of Rad21 at *CASCADES* enhancer and Sox2 proximal promoter facilitates chromatin looping. This allows for RNA Pol II binding at those sites and for simultaneous transcription of *CASCADES* and Sox2. The *CASCADES* transcript acts as a “TF‐trapper” and entraps YY1 at the proximal promoter of Sox2 and allows for continuous transcription of Sox2 in a positive feedback loop. (B) The CSC hypothesis posits that within GBM, there is a heterogenous tumor cell population. Conventional cancer therapies target actively dividing cells, whereby CSCs that are capable of becoming quiescent can escape the cytotoxic effects and can undergo self‐renewal and promote tumor relapse. Therapies that specifically target the CSC population can lead to tumor regression. Antisense oligo therapy targeting a cell‐ and cancer‐specific lncRNA, such as *CASCADES*, can further enhance the effects of anti‐CSC therapy, and not only lead to tumor shrinkage, but also prevent tumor relapse.

It has previously been reported that in mouse embryonic stem cells, super‐enhancers associated with *SOX2* contribute to 90% of its expression [66]. Furthermore, in ESCs, co‐occupancy of master transcription factors, such as Oct4 (POU5F1), Sox2, and Nanog, along with exceptionally high levels of H3K27ac and DNase I hypersensitivity, is highly representative of super‐enhancer activity [67, 68]. Our data shows that the *CASCADES* genomic locus, which contains the SE for *SOX2*, has high occupancy of Nanog and POU5F1 at the hub enhancer, along with other important TFs, such as RBBP5, Tcf12, and ZNF143. Additionally, we found that YY1 binds the hub enhancer element as well as the *SOX2* proximal promoter and that YY1 binding is reduced upon knockdown of *CASCADES*. YY1, also known as Yin Yang 1, has previously been shown to mediate physical enhancer‐promoter interactions involving super‐enhancers [69, 70]. Our findings support a model in which binding of Rad21 at the *CASCADES* hub enhancer element and *SOX2* proximal promoter could facilitate chromatin looping. This model posits that such looping might help in the recruitment of RNA Pol II binding at those sites, thereby promoting simultaneous transcription of *CASCADES* and *SOX2*. The *CASCADES* transcript then acts as a “transcription factor‐trapper” to entrap YY1 at the proximal promoter of *SOX2* and allow for continued transcription of Sox2 in a positive feedback loop (Fig. 6A). However, further experimental validation, such as targeted mutations or deletions within the CASCADES sequence to demonstrate disruption of this loop is required to definitively establish this mechanism. Taken together, our data show that *CASCADES* is transcribed from a locus that acts as a super‐enhancer for *SOX2*, and that *CASCADES* transcript itself modulates the activity of this SE in a positive feedback loop to promote the expression of *SOX2*.

In summary, *CASCADES* is a novel super‐enhancer‐associated lncRNA that modulates *SOX2* expression and thereby regulates the GSC phenotype, in a manner analogous to other SE‐associated lncRNAs, such as CCAT1‐L [71], *CARMEN* [72], and LINC01503 [73], which have cell type‐specific functions in maintaining cell identity and homeostasis. Our data show that the knockdown of *CASCADES* has global effects on cell cycle pathways, specifically in GSCs, and promotes GSC differentiation towards a neuronal phenotype, identifying *CASCADES* as a target to disrupt the GSC identity in glioblastoma (Fig. 6B).

## 5 Conclusion

The importance of the CSC subpopulation in tumor progression and treatment resistance has been demonstrated in multiple hematologic and solid cancers, including glioblastoma. It has been hypothesized that the effective treatment of glioblastoma will require the development of new therapies that are capable of directly targeting GSCs. Our work identifies *CASCADES* as a novel super‐enhancer‐associated lncRNA that modulates *SOX2* expression in GSCs and thereby maintains the cancer stem cell identity in glioblastoma. By targeting a cancer‐specific gene, such as *CASCADES*, we can directly target and disrupt the GSC identity in glioblastoma and potentially overcome CSC‐mediated treatment resistance.

## Conflict of interest

The authors declare no conflict of interest.

## Author contributions

US, CL, JJW, and SD contributed to the conception and design of the work. SD provided overall supervision. All authors contributed to data collection/analysis/interpretation in some capacity, with US, MN, CL, MJ, JKW, NS, FSV, AR, PMK, SK, CAS, JK, JJW, RGWV, MG and JTR substantially contributing to the collection, analysis and interpretation of data. US, MN, MJ, SK, CAS, JK, JKW, RGWV, MG, JTR and SD substantially drafted and revised the work. All authors reviewed the manuscript.

## Supporting information


**Fig. S1.** Validation of *CASCADES* as a transcribed gene.
**Fig. S2.** *CASCADES* is expressed across various tissues in the body and TCGA data indicate high *CASCADES* expressors with gliomas experience a survival disadvantage.
**Fig. S3.** *CASCADES* expression was knockdown using siRNA designed against the *CASCADES* transcript in several cell lines.
**Fig. S4.** The glioma stem cell line Gli489 (N = 3/group) was treated with siRNA directed against CASCADES or universal negative control (scrambled) for 72 h.
**Fig. S5.** The glioma stem cell line GliNS1 (N = 3/group) was treated with siRNA directed against CASCADES or universal negative control (scrambled) for 72 h.
**Fig. S6.** The fetal neural stem cell line HFNS7450 (N = 3/group) was treated with siRNA directed against CASCADES or universal negative control (scrambled) for 72 h.
**Fig. S7.** The human fetal neural stem cell line HFNS 6562 (N = 3/group) was treated with siRNA targeting against CASCADES or universal negative control (scrambled) for 72 h.
**Fig. S8.** The CASCADES expression was assessed in different cellular fractions and compared to total RNA.
**Fig. S9.** Chromatin immunoprecipitation was performed for Rad21, YY1, and RNA Pol II. H3 served as control.
**Fig. S10.** The CASCADES expression was knocked down using antisense oligonucleotides designed against the CASCADES transcript or the enhancer element found within the transcript.

**Table S1.** Primers.
**Table S2.** Top 20 lncRNA targets.

## Data Availability

All raw and processed sequencing data generated in this study have been submitted to the NCBI Gene Expression Omnibus (GEO; https://www.ncbi.nlm.nih.gov/geo/) under accession number GSE GSE157506.
