# Supplementary material for: CASCADES, a novel SOX2 super‐enhancer‐associated long noncoding RNA, regulates cancer stem cell specification and differentiation in glioblastoma
Source: Mol Oncol. 2024 Sep 25;19(3):764–84. doi: 10.1002/1878-0261.13735 (PMC11887672; doi:10.1002/1878-0261.13735)
Supplement: Supplementary file 1 — Fig. S1. Validation of CASCADES as a transcribed gene. Fig. S2. CASCADES is expressed across various tissues in the body and TCGA data indicate high CASCADES expressors with gliomas experience a survival disadvantage. Fig. S3. CASCADES expression was knockdown using siRNA designed against the CASCADES transcript in several cell lines. Fig. S4. The glioma stem cell line Gli489 (N = 3/group) was treated with siRNA directed against CASCADES or universal negative control (scrambled) for 72 h. Fig. S5. The glioma stem cell line GliNS1 (N = 3/group) was treated with siRNA directed against CASCADES or universal negative control (scrambled) for 72 h. Fig. S6. The fetal neural stem cell line HFNS7450 (N = 3/group) was treated with siRNA directed against CASCADES or universal negative control (scrambled) for 72 h. Fig. S7. The human fetal neural stem cell line HFNS 6562 (N = 3/group) was treated with siRNA targeting against CASCADES or universal negative control (scrambled) for 72 h. Fig. S8. The CASCADES expression was assessed in different cellular fractions and compared to total RNA. Fig. S9. Chromatin immunoprecipitation was performed for Rad21, YY1, and RNA Pol II. H3 served as control. Fig. S10. The CASCADES expression was knocked down using antisense oligonucleotides designed against the CASCADES transcript or the enhancer element found within the transcript. [file MOL2-19-764-s001.pdf]

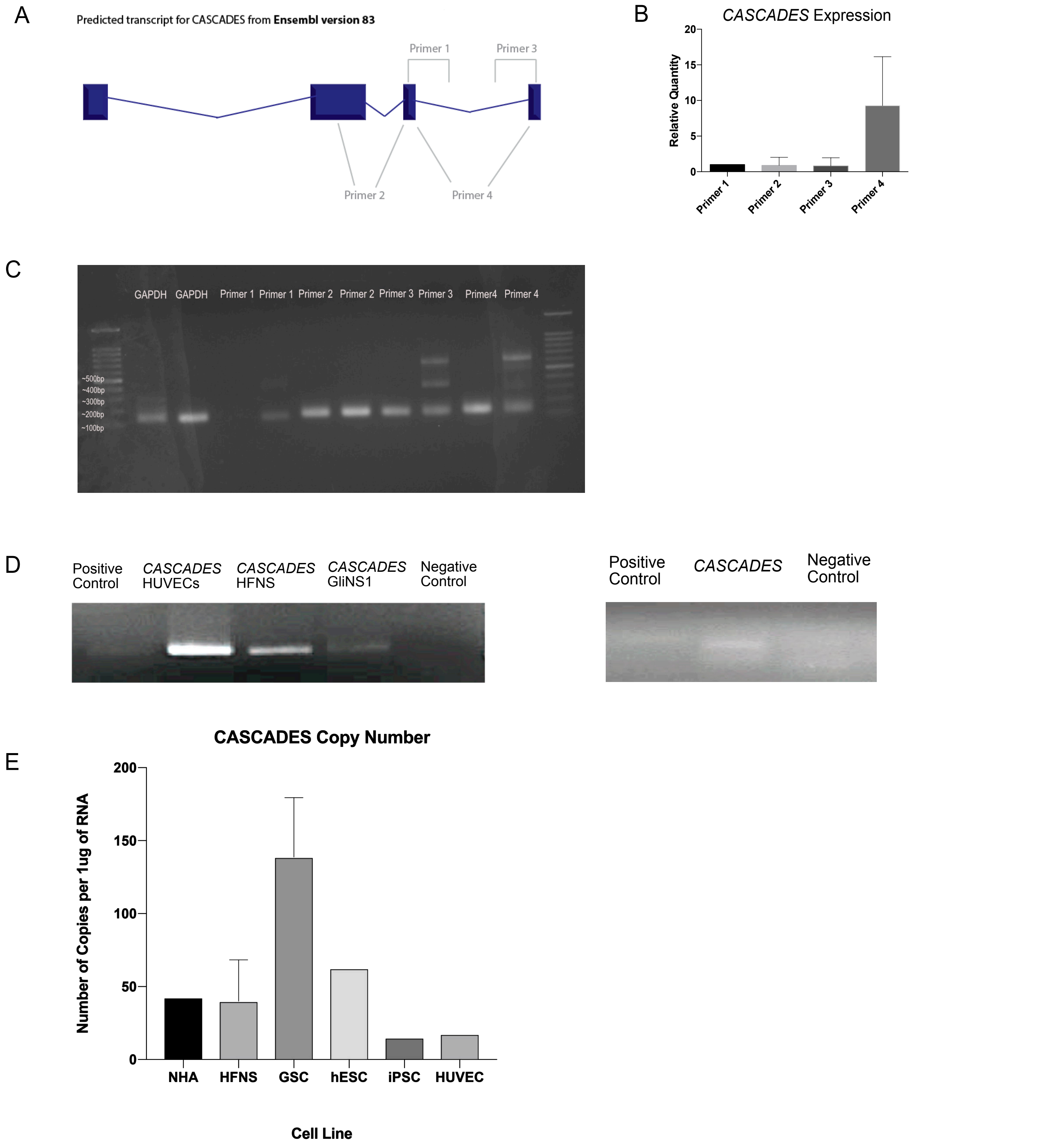

Supplementary Figure 1. A, B. The expression of *CASCADES* upon primer crawling designed across different areas of the first transcript. **C.** 3'Rapid Amplification of cDNA Ends (RACE) of *CASCADES* in glioma stem cells (GliNS1), normal human fetal neural stem cells (HFNS), and human umbilical vein endothelial cells (HUVECs). **D.** 5'RACE of *CASCADES* in glioma stem cells (GliNS1) cell line. **E.** The copy number of *CASCADES* across different cell lines.

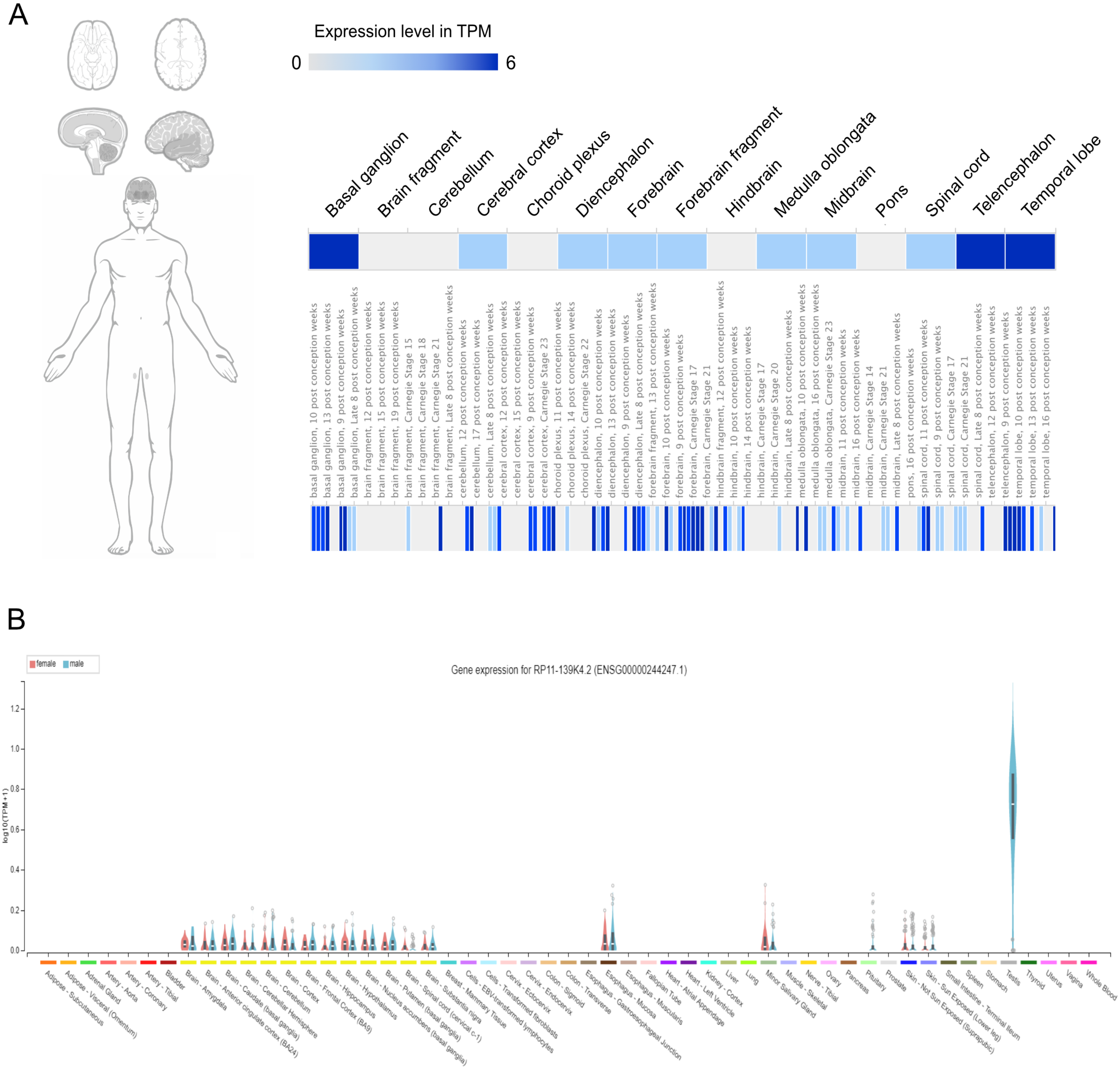

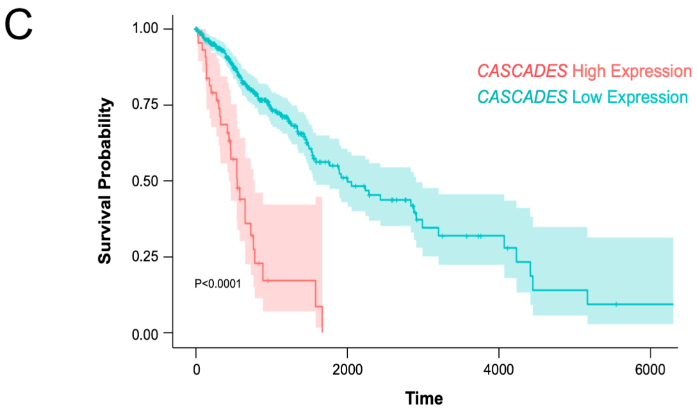

Supplementary Figure 2. A, B. EMBL-EBI Expression Atlas profile of *CASCADES* in the different body tissues and the developing brain. **C.** Patient survival data from The Cancer Genome Atlas (TCGA) shows a survival disadvantage for glioma patients with high expression of *CASCADES* (P<0.0001).

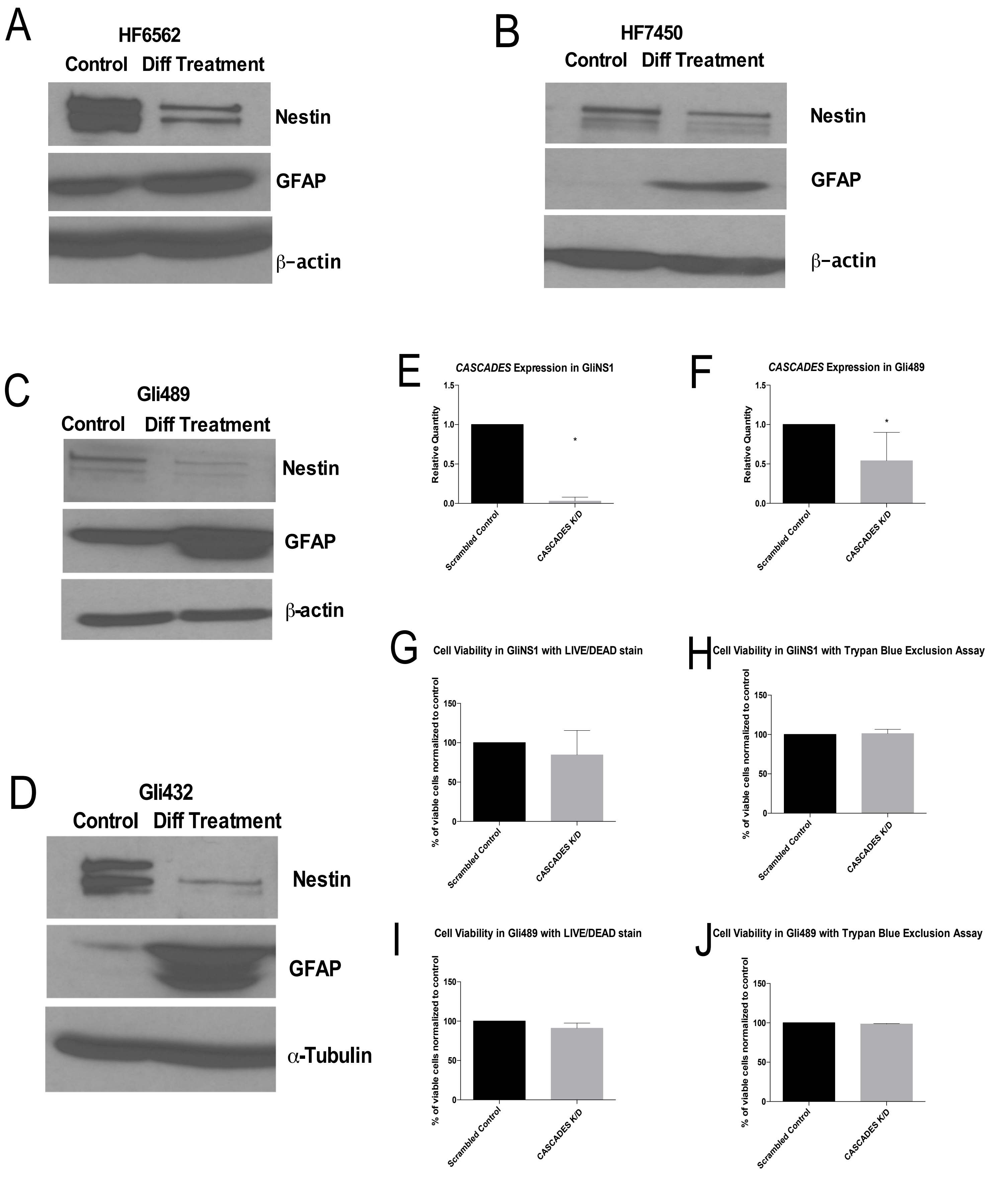

Supplementary Figure 3.

Two HFNS cell lines, HF6562 (A) and HF7450 (B) as well as two GSC lines, Gli489 (C) and Gli432 (D) were forced to differentiate into a strocytes. The expression of neural stem cell marker, Nestin had decreased upon forced differentiation, while expression of glial fibrillary acidic protein (GFAP) increased.

The *CASCADES* expression was knockdown using siRNA designed against the *CASCADES* transcript. The expression of *CASCADES* was significantly decreased in both GliNS1 (E) and Gli489 (F) cell lines. Furthermore, the knockdown of *CASCADES* did not adversely affect cell viability in either GliNS1 (G,H), or Gli489 (I, J), as assessed by both LIVE/DEAD staining and Trypan Blue Exclusion assay.

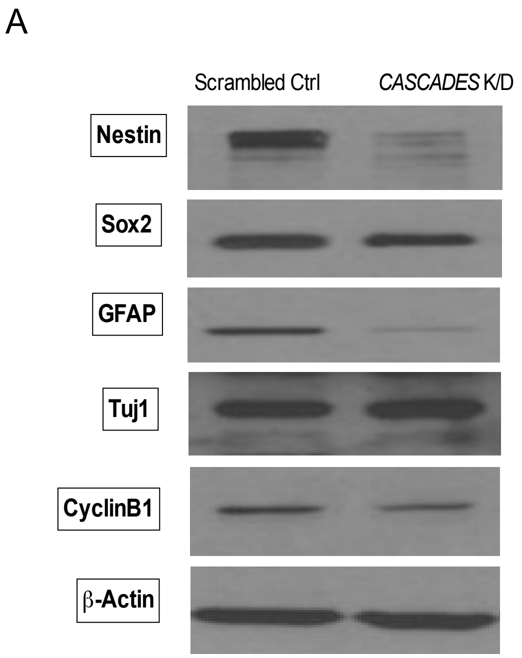

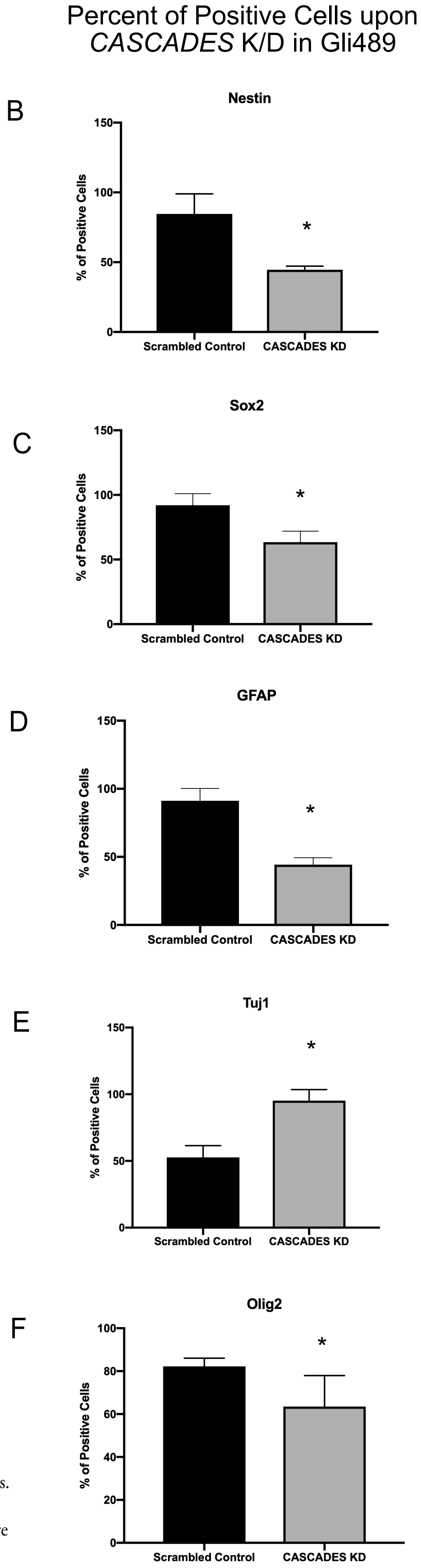

Supplementary Figure 4. he glioma stem cell line Gli489 (N=3/group) was treated with siRNA directed against *CASCADES* or universal negative control (scrambled) for 72 hours.

A. The Western blotting analysis of various neural stemness markers in *CASCADES* K/D vs. scrambled control

B-F. The percentage of positive cells out of total cells identified through confocal microscopy were plotted as bar graphs.

CASCADES Knockdown in GliNS1

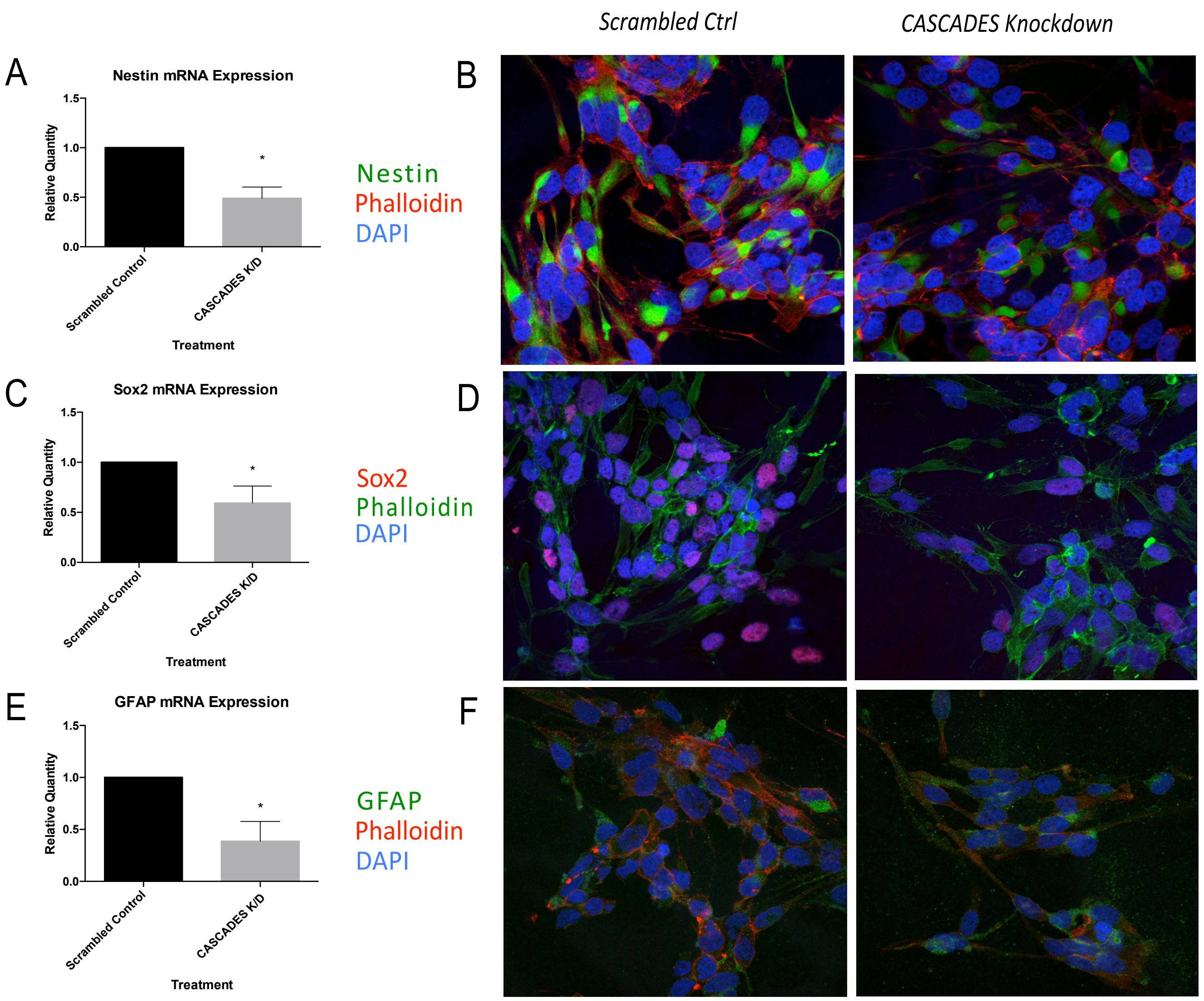

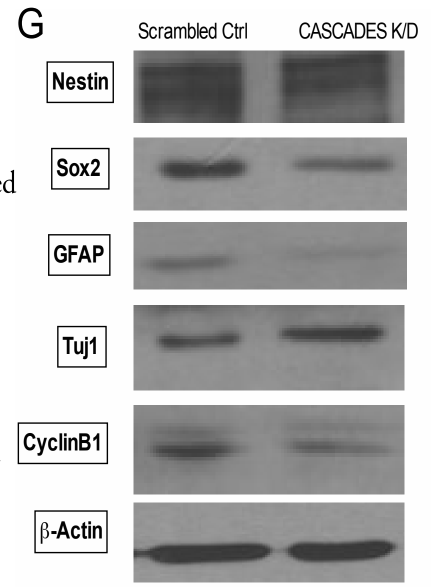

Supplementary Figure 5. The glioma stem cell line GliNS1 (N=3/group) was treated with siRNA directed against *CASCADES* or universal negative control (scrambled) for 72 hours.

The stemness markers, Nestin (A,B,&G) and Sox2 (C,D,&G) were significant decreased after *CASCADES* knockdown, compared to scrambled controls. Furthermore, the expression of GFAP, an astrocyte marker, also showed a decrease (E,F,&G). In comparison, the differentiated neuron marker, Tuj1 was greatly increased in expression, after *CASCADES* knockdown. Furthermore, the knockdown of *CASCADES* decreased the expression of cyclin B1 (G), compared to scrambled controls.

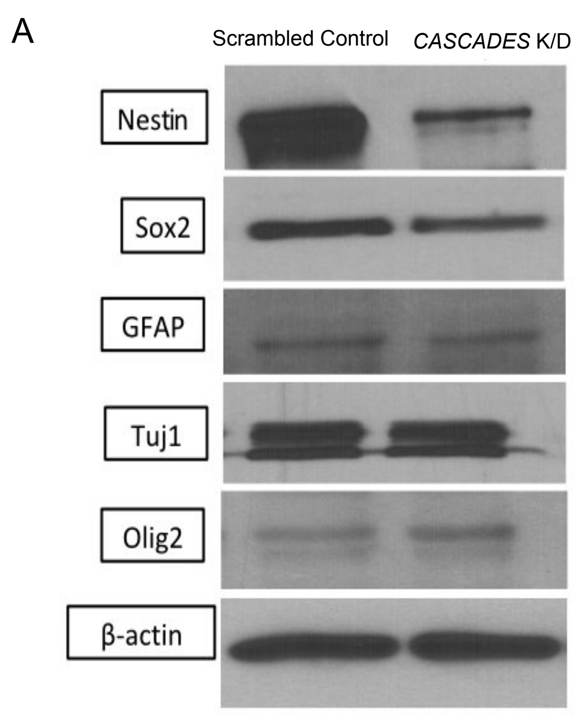

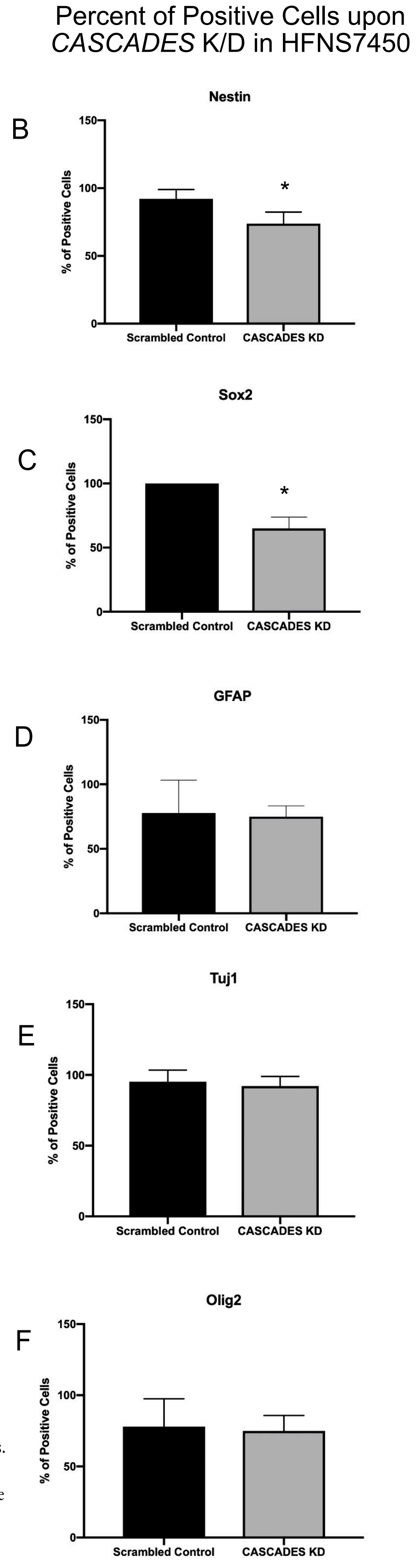

Supplementary Figure 6. he fetal neural stem cell line HFNS7450 (N=3/group) was treated with siRNA directed against *CASCADES* or universal negative control (scrambled) for 72 hours.

A. The Western blotting analysis of various neural stemness markers in *CASCADES* K/D vs. scrambled control

B-F. The percentage of positive cells out of total cells identified through confocal microscopy were plotted as bar graphs.

CASCADES Knockdown in HFNS 6562

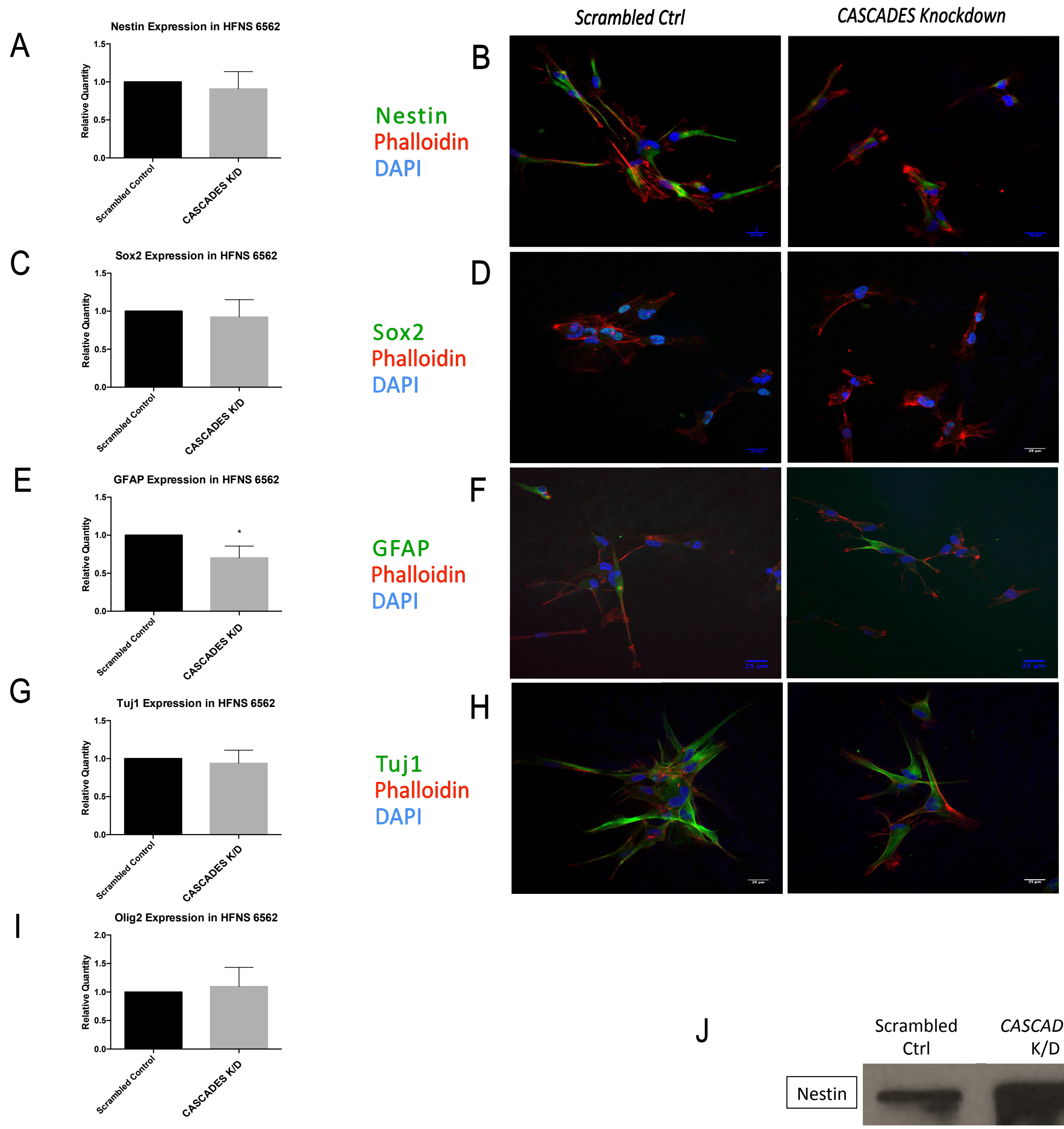

J

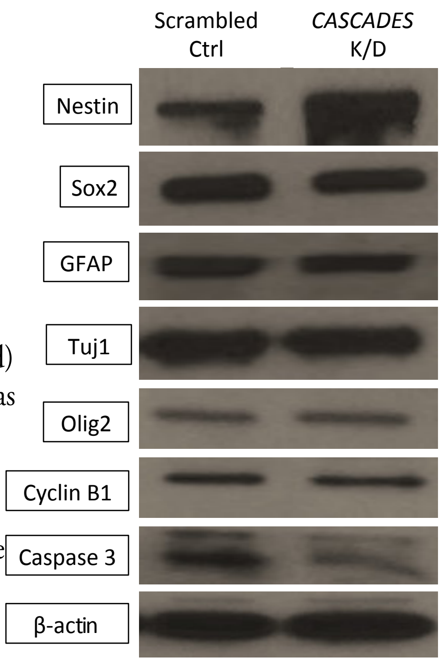

Supplementary Figure 7. The human fetal neural stem cell line HFNS 6562 (N=3/group) was treated with siRNA targeting against *CASCADES* or universal negative control (scrambled) for 72 hours. The stemness marker, Nestin (B&J) showed slight increase in expression, whereas Sox2 (D&J) decreased after CASCADES knockdown, compared to scrambled controls. Furthermore, the expression of GFAP, an astrocyte marker, also showed a decrease (F&J). In comparison, Tuj1 (H&J), Olig2 (J), and cyclin B1 (J), did not show significantly different expression after CASCADES knockdown when compared to scrambled controls. Furthermore no statistically significant differences were detected at the mRNA level (A,C,E,&G).

CASCADES Localization Controls

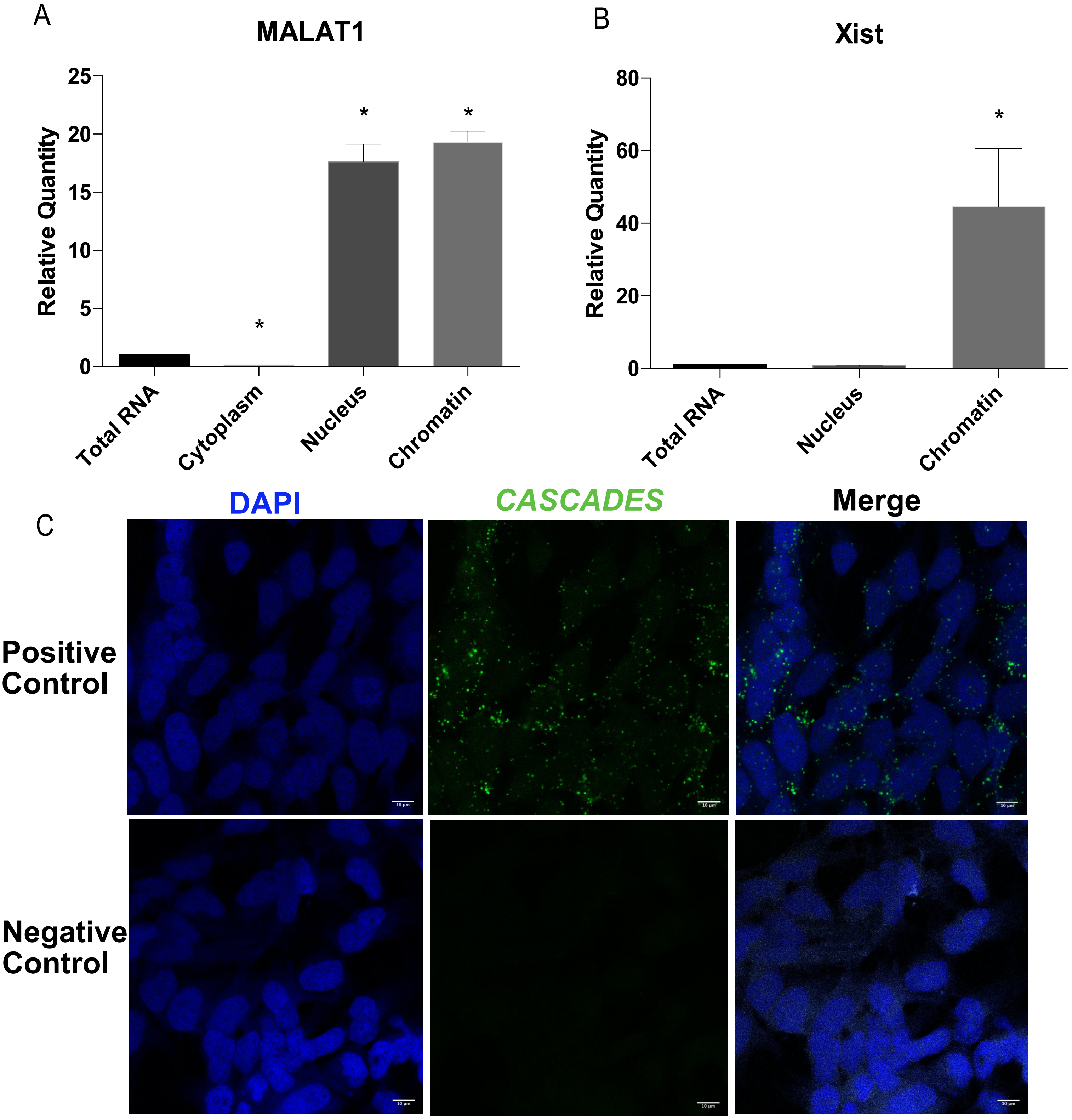

Supplementary Figure 8. The *CASCADES* expression was assessed in different cellular fractions and compared to total RNA.(A) MALAT1 (B) and Xist served as controls.
(C) The positive and negative controls for the RNA *in situ* hybridization.

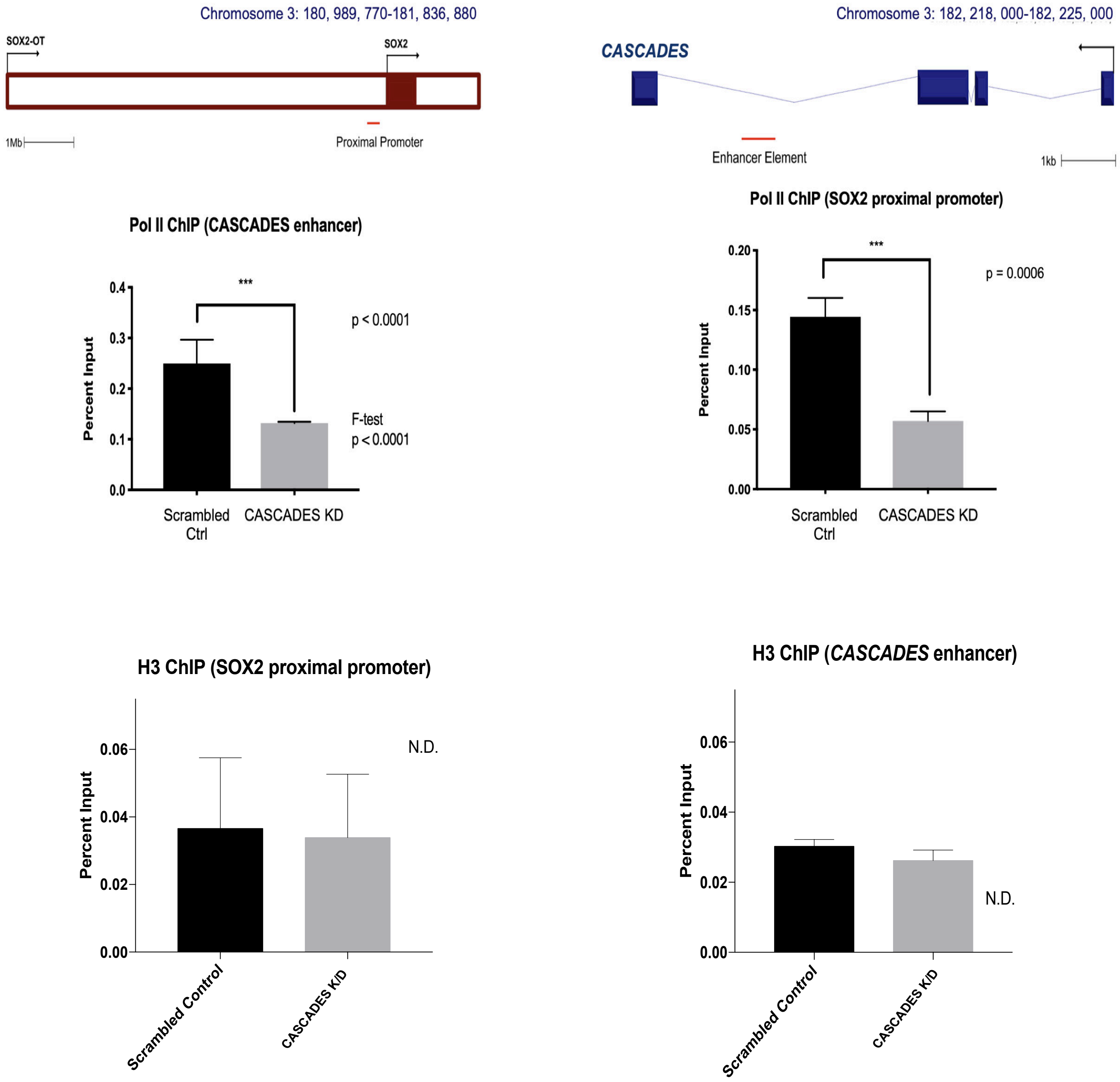

Supplementary Figure 9. Chromatin immunoprecipitation was performed for Rad21, YY1, and RNA Pol II. H3 served as control.

CASCADES Knockdown using ASO

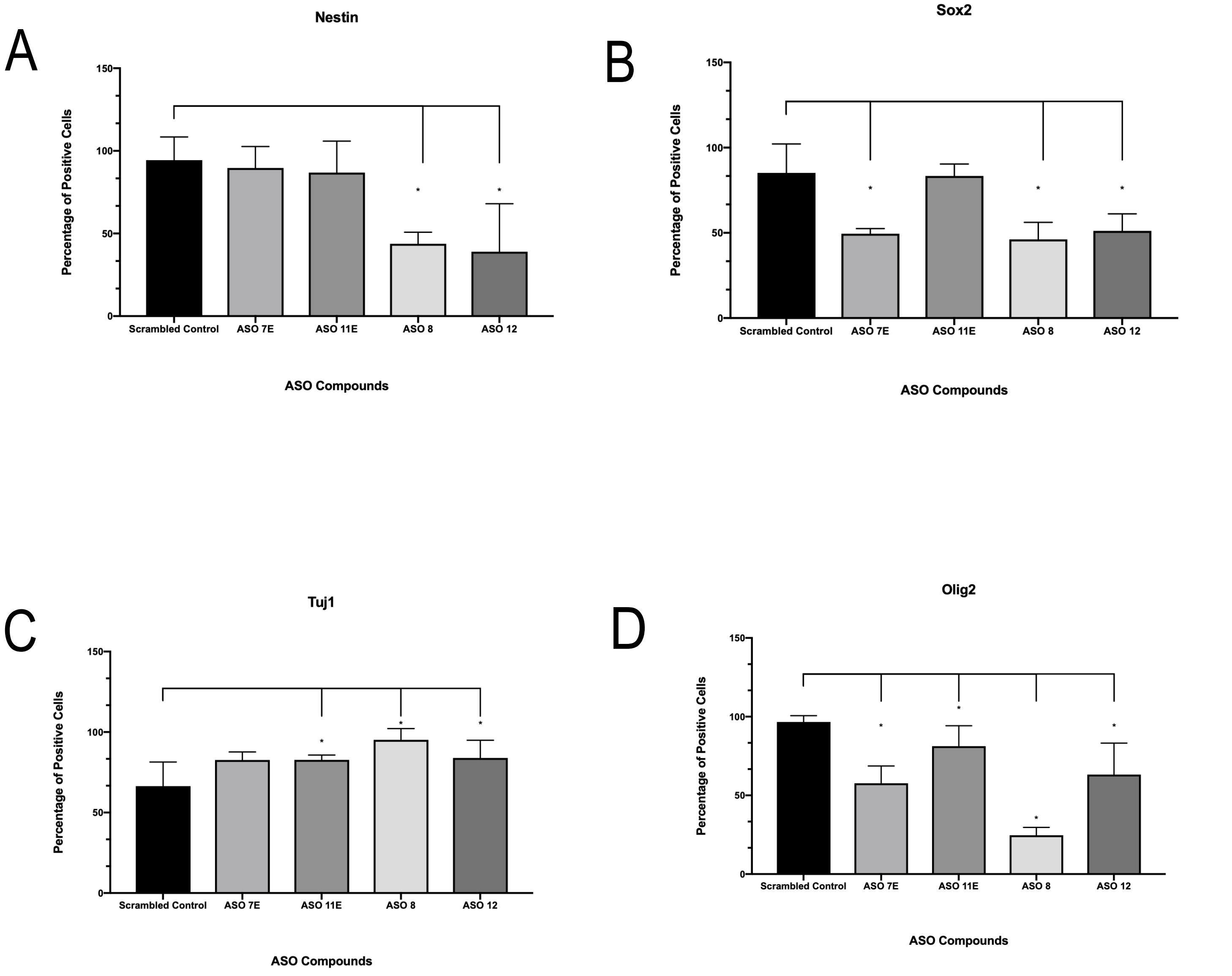

Supplementary Figure 10. The *CASCADES* expression was knocked down using antisense oligonucleotides designed against the *CASCADES* transcript or the enhancer element found within the transcript.

The knockdown of *CASCADES* using the ASO 8 & ASO 12 resulted in significant reduction of percentage of cells expressing Nestin (A), Sox2(B), and Olig2 (D), and an increase in Tuj1 (C).
