# Supplementary material for: CASCADES, a novel SOX2 super‐enhancer‐associated long noncoding RNA, regulates cancer stem cell specification and differentiation in glioblastoma
Source: Mol Oncol. 2024 Sep 25;19(3):764–84. doi: 10.1002/1878-0261.13735 (PMC11887672; doi:10.1002/1878-0261.13735)
Supplement: Supplementary file 2 — Table S1. Primers. Table S2. Top 20 lncRNA targets. [file MOL2-19-764-s002.pdf]

**Supplementary Table 1 – Primers `** `

**GAPDH PRIMERS**	
**FORWARD**	AGATCCCTCCAAAATCAAGTGG
**REVERSE**	GGCAGAGATGATGACCCTTTT
***CASCADES* PRIMERS**	
**FORWARD**	TGCGAAAACAGAAGCTGAGGA
**REVERSE**	GCCGCTTGTCAGAAGAAACTC
***CASCADES* – SIRNA**	
**SENSE**	rCrArC rArGrC rArUrA rCrCrU rUrGrG rUrUrA rUrUrC rUrCrA rUrUrU
**ANTI-SENSE**	rArUrG rArGrA rArUrA rArCrC rArArG rGrUrA rUrGrC rUrGT G
**SENSE**	rArUrA rCrArA rArCrU rCrCrC rUrGrC rUrGrU rCrArU rGrArA rGrCrU
**ANTI-SENSE**	rCrUrU rCrArU rGrArC rArGrC rArGrG rGrArG rUrUrU rGrUA T
**SENSE**	rCrArA rGrGrC rUrGrU rCrCrC rUrUrC rCrArU rCrUrG rArArG rGrCrU
**ANTI-SENSE**	rCrCrU rUrCrA rGrArU rGrGrA rArGrG rGrArC rArGrC rCrUT G
**5'RACE PRIMERS**	
**5N1**	TTC TCT TTG GCT CCT TGG CAC
**5N2**	TCG CTT TTC TTG CTC TCC TGC
**5N3**	TCC CGA TGG TTT GCT CTA CAA
**5N4**	TTA TCC AGG ATG AGC GGG TGT
**3'RACE PRIMERS**	
**3N1**	TGG AAA GAA GGC AGG AGA GCA
**3N2**	TCT CCA GAC CAG TGC CAA GGA
***XIST* PRIMERS**	
**FORWARD**	GGG CCT TGG AGG GAA ACA GT
**REVERSE**	TGC CCT AGA CAA GGA TGC CC
***MALAT1* PRIMERS**	
**FORWARD**	GAC GGA GGT TGA GAT GAA GC
**REVERSE**	ATT CGG GGC TCT GTA GTC CT
***DCUN1D1* PRIMERS**	
**FORWARD**	CAA CTG GTT CTG TCC GGT GA
**REVERSE**	GAC TGC AAT GAA TGT CCA TGC C
***SOX2-OT* PRIMERS**	
**FORWARD**	AGC CGA AAT GGA TTC ACG GT

**REVERSE**	GAC TGC AAT GAA TGT CCA TGC C
**PRIMER CRAWLING**	
**PAIR 1**	
**FORWARD**	TCC CTA CCC GGT GAA CTT TG
**REVERSE**	CTC TTT GGC TCC TTG GCA CTR
**PAIR 2**	
**FORWARD**	CAT GGC CAT ATT GTA GAG CAA ACC
**REVERSE**	GAC GCA CGT GAT AAA GGC TGA
**PAIR 3**	
**FORWARD**	TGC GAA AAC AGA AGC TGA GGA
**REVERSE**	GCC GCT TGT CAG AAG AAA CTC

Gene Symbol	Nearby Gene	Strand	Location	Splice Variants	Longest Transcript Length
**XXbac-BPG27H4.8**	Oct3/Oct4(POU5F1)	Reverse	chrom 6	1	799
**HCG20**	Oct3/Oct4(POU5F1)	Forward	chrom 6	2	1202
**XXbac-BPG181B23.7**	Oct3/Oct4(POU5F1)	Reverse	chrom 6	1	1207
**LINC01149**	Oct3/Oct4(POU5F1)	Forward	chrom 6	1	2141
**RP11-139K4.2**	Sox2	Reverse	chrom 3	1	621
**RP11-416O18.2**	Sox2	Forward	chrom 3	1	477
**CASC11**	Myc & POU5F1B	Reverse	chrom 8	3	3301
**CASC19**	Myc & POU5F1B	Reverse	chrom 8	1	367
**PCAT1**	Myc & POU5F1B	Forward	chrom 8	2	1992
**RP11-761N21.1**	Beta-Catenin (CTNNB1)	Forward	chrom 3	1	500
**RP11-391M1.4**	Beta-Catenin (CTNNB1)	Forward	chrom 3	1	2122
**LINC01060**	Rex1 (Zfp42)	Forward	chrom 4	6	2435
**AE000658.31**	Sall2	Reverse	chrom 14	1	570
**RP11-998D10.7**	Sall2	Forward	chrom 14	1	419
**AL161668.5**	Sall2	Forward	chrom 14	2	478
**RP11-219E7.4**	Sall2	Reverse	chrom 14	1	351
**RP11-219E7.3**	Sall2	Reverse	chrom 14	2	549
**LINC00945**	Olig1/Olig2	Forward	chrom 21	1	753
**AP000289.6**	Olig1/Olig2	Reverse	chrom 21	1	1361
**AP000569.9**	Olig1/Olig2	Reverse	chrom 21	1	591

Supplementary Table 2: Top 20 lncRNA targets.
